# Distinct space-use patterns are associated with alternative reproductive tactics in lekking ruffs *Calidris pugnax*

**DOI:** 10.1098/rsos.262020

**Published:** 2026-11-11

**Authors:** Giovanni Spezie, Gergely Dinya, Anna Gelencsér-Horváth, Wolfgang Goymann, Leonida Fusani, Clemens Küpper

**Affiliations:** ^1^ Department of Interdisciplinary Life Sciences, Konrad Lorenz Institute of Ethology, University of Veterinary Medicine, Vienna, Austria; ^2^ Research Group Behavioural Genetics and Evolutionary Ecology, Max Planck Institute for Biological Intelligence, Seewiesen, Germany; ^3^ Faculty of Informatics, Eötvös Loránd University, Budapest, Hungary; ^4^ Faculty of Information Technology and Bionics, Pázmány Péter Catholic University, Budapest, Hungary; ^5^ Department of Behavioral Neurobiology, Max Planck Institute for Biological Intelligence, Seewiesen, Germany; ^6^ Department Biologie II, Ludwig Maximilian University of Munich, Planegg-Martinsried, Germany; ^7^ Department of Behavioural and Cognitive Biology, University of Vienna, Vienna, Austria

**Keywords:** alternative reproductive tactics, lekking, spatial ecology, testosterone, behavioural plasticity

## Abstract

Territoriality is a key strategy through which animals secure access to reproductive opportunities, yet in many lekking species, males differ in their investment in defending space. Breeding aggregations often include territorial residents and non-territorial floaters, raising questions about the flexibility and endocrine regulation of alternative spatial tactics. The ruff (*Calidris pugnax*) provides a powerful model to address these questions because males occur as three genetically determined reproductive morphs: territorial independents and two non-territorial floater morphs, satellites and faeders. Using artificial intelligence-assisted tracking in a captive colony, we quantified fine-scale space-use patterns within experimental and control leks. Our aim was to test whether male morphs adjust spatial behaviour when social opportunities arise. Independents exhibited smaller and more segregated activity areas, whereas satellites and faeders showed larger, overlapping activity areas, consistent with a floater strategy. Despite experimentally manipulating lek composition to create single-morph social environments, neither satellites nor faeders adopted territorial-like spatial behaviour. In addition, circulating concentrations of testosterone were higher in independents than faeders and satellites, but short-term variation in testosterone was not linked to changes in spatial behaviour and experimental treatment. These findings support the view that space-use tactics in ruffs represent fixed specializations rather than flexible responses to social opportunities.

## 1. Introduction

Territoriality is a widespread behavioural strategy by which individuals secure access to critical resources necessary for reproduction. In non-resource-based polygynous mating systems, such as classical leks, territories are devoid of any resources other than visiting mates, therefore territoriality reflects competition for position and dominance. For example, central positions within leks are often associated with higher mating success [1–3] and courting males are under strong selective pressures for investing in territory attendance, site fidelity and courtship [4,5]. Yet in many lekking species, individuals of the same sex do not invest equally in defending space. Instead, aggregations of breeding males commonly include a mixture of territorial ‘residents’ and non-territorial ‘floaters’, defined as individuals that move dynamically both within and between display arenas without maintaining a territory of their own [6,7]. In manakins, for example, floater males visit multiple display arenas during the breeding season, display cooperatively with resident ‘alphas’ and forego reproduction [8,9]. Similarly, in some lekking cichlids, non-territorial ‘sneaker’ males obtain copulations opportunistically by exploiting the investment of territorial males [10,11].

The presence of floaters raises key questions about the function and flexibility of different territorial strategies. Floaters may simply represent competitively inferior individuals (i.e. younger or less experienced) that cannot secure and maintain a territory, therefore following a *conditional* alternative reproductive tactic (ART) that reflects their lower resource-holding potential. In other species, however, floating behaviour appears to be a *fixed* tactic, namely a discrete, genetically or developmentally determined ART that is part of a behavioural polymorphism maintained by frequency-dependent selection [12,13]. In such cases, floater males typically exhibit suites of morphological, physiological and behavioural traits adapted to a non-territorial or sneaker mating role, such as a smaller body size and a lack of weapons or ornaments [11,14,15].

Despite the central role that spatial behaviour plays in characterizing the behavioural strategies of different conditional or fixed ARTs, most work on space use in lekking animals has focused on differences in large-scale movement patterns between residents and floaters across leks, for example, using satellite telemetry [16–19]. Fewer studies have systematically tracked movement patterns at smaller scale (i.e. within leks) to determine how floaters position themselves relative to territorial males, how consistent their space use is over time or whether they adjust their movements when the social environment changes (e.g. [20]). Likewise, the mechanisms underlying differences between and within males and shifts in space-use strategies are poorly understood. Removal experiments of territorial males—critical for testing tactic flexibility in floaters and residents—have been conducted only in a limited number of lekking species because they are logistically challenging and require long-term behavioural monitoring ([21–23]; see also [24]). As a result, fundamental questions persist: how do floaters navigate the spatial and social structure of leks? And under what conditions, if any, can floaters transition into territorial roles?

Furthermore, prior work suggests that spatial tactics in lekking animals are closely linked to differences in endocrine state, particularly circulating testosterone, the key androgen hormone associated with reproductive behaviour [25–28]. Floaters typically exhibit lower baseline androgen levels than dominant territory holders, consistent with reduced aggression and absence of territoriality [29–32]. Yet, it remains unclear whether these hormonal differences reflect fixed, tactic-specific constraints or whether testosterone levels are modulated plastically when social opportunities arise. According to the ‘challenge hypothesis’ [33], testosterone levels rise in response to social instability and male–male competition, facilitating aggressive behaviour and territory defence ([34,35]; but see [36]). Some studies have tracked endocrine state before and after experimentally induced status changes via removal experiments, or conversely, have investigated shifts in territorial behaviour after experimental administration of androgens. In both cases, the results are mixed: in some species, elevation to territorial status triggered a rise in testosterone [26,37,38] and testosterone implants in floater males activated the full suite of territorial and courtship behaviours [39–41], whereas in others, floater males maintained low androgen profiles even when social conditions changed [25] and did not fully switch to territorial behaviours after androgen administration [42,43]. Thus, whether hormonal profiles constrain tactic flexibility across lekking species remains an open question. In particular, if circulating testosterone mediates adjustments in territorial behaviour, exposing males to experimentally altered social environment may induce coordinated changes in both testosterone concentrations and space-use patterns.

Ruffs (*Calidris pugnax*) are lekking shorebirds that feature discrete, fixed ARTs that differ in the extent of territoriality, suggesting different requirements for space use. ‘Independents’ display to visiting females on small territories within lekking sites, which are fiercely defended against competitors; ‘satellites’ lack territorial behaviour, but associate with independents on their territories, performing cooperative co-displays as subordinated partners; last, ‘faeders’ are non-ornamented female mimics, smaller in size and behave like sneakers [44–47]. The latter two morphs—representing, respectively, approximately 10%–20% and approximately 1%–2% of ruffs in wild populations [44,48,49]—therefore qualify as floaters, as they do not invest in territory defence but instead associate with independents and/or follow female movements to seek mating opportunities [49,50]. Yet, a fraction of satellite males (called ‘central’) exhibit a greater degree of lek attachment than ‘peripheral’ satellites [51,52], and while ‘resident’ independents are typically territorial and site-faithful during the breeding season, young and/or low-quality independents (called ‘marginals’) are unable to secure a territory on leks and follow a conditional, reversible, floater-like strategy, probably linked to their lower resource holding potential [49,50,52]. Genomic work has shown that the satellite and faeder phenotypes are associated with a dominant autosomal supergene that arose through chromosomal inversion and that underlies suites of morphological, physiological and behavioural differences among morphs [46,47,53]. Thus, the existence of clear morph categories—and the presence of nested conditional tactics within fixed morphs—makes the ruff an ideal species to investigate spatial patterns associated with different morphs and to ask whether individuals can plastically adjust spatial strategies in response to social opportunity. A recent telemetry study revealed that large-scale movement patterns between breeding sites are more dynamic across all morphs than previously acknowledged [17]. However, experimental assessments of plastic responses are lacking in ruffs, primarily because of the rarity of floater morphs, and the highly dynamic spatial behaviour of males across morphs in the wild.

In this study, we quantified tactic-specific space-use patterns and their hormonal correlates in lekking ruffs to test whether genetically determined ARTs retain behavioural plasticity when the social environment is experimentally altered. Using artificial intelligence (AI)-assisted tracking, we quantified individual movements within leks with high temporal and spatial resolution across the whole lekking season. Studying ruffs under controlled aviary conditions allowed us to experimentally manipulate lek composition and create vacant social niches by removing males of specific morphs. Our aims were to: (i) characterize individual variation and differences in space use among independents, satellites and faeders, and determine how these spatial patterns relate to testosterone levels throughout the breeding season; (ii) determine how spatial behaviour relates to mating success in different morphs by mapping copulations relative to male activity areas within leks; and (iii) experimentally test plasticity in the two floater morphs (faeders and satellites) via controlled exclusion of independent males from experimental leks. More specifically, we asked whether (i) inter-individual distance, size and overlap of activity areas differ between independents, satellites and faeders, consistent with territorial and floater behaviour, respectively, and whether within-season fluctuations in those measures co-varies with testosterone levels within and between morphs; (ii) whether the spatial relationship between copulation locations and male activity areas differs among morphs and changes following experimental manipulation of the social environment, indicating whether mating behaviour remains associated with morph-specific patterns of space use; and (iii) floater morphs alter space-use strategies when dominant independents are removed. Together, these approaches allowed us to connect genetic, endocrinological and behavioural perspectives on ARTs and to test whether tactic-specific space-use strategies are strictly fixed or sensitive to variation in the social environment.

## 2. Material and methods

### 2.1. Study subjects and experimental design

The study was conducted using ruffs from a captive flock housed at the Max Planck Institute for Biological Intelligence in Seewiesen, Germany. The flock contains all three morphs and was established from different sources of captively bred ruffs (for details, see [45,54]). Sex and morph are identified through genetic markers within weeks after hatching [55]. During the breeding season, males of this flock exhibit the typical repertoire of courtship postures and the agonistic and cooperative interactions including co-display observed in nature [44,48,51].

The experiment was run over two breeding seasons (May–June 2023 and 2024). In both years, the inner part of an aviary was divided into five experimental units (hereafter ‘units’) consisting of 4 × 5 m arenas with natural grass and two water pools; each unit was connected to adjacent separate indoor rooms (2 × 5 m) where the experimental males were housed overnight (figure 1A). Units were separated by tarpaulin screens to avoid visual contact between adjacent arenas. Acoustic isolation was not implemented as courtship displays and agonistic interactions do not include vocalizations in ruffs [56]. Each unit housed 15–16 females, which remained continuously present within the experimental units for the entire duration of the lekking season. We arranged male morphs in three units to create artificial leks consisting only of one morph (‘single-morph’ units: one unit with five independents, one unit with five satellites, one unit with five faeders), while the other two ‘mixed-morph’ units contained a combination of all morphs (three independents, one faeder and one satellite; figure 1A). Thus, the experiment consisted of three single-morph units (*n* = 2 replicate units per treatment across years, 10 males in total per morph) and two mixed-morph units (*n* = 4 replicate units across years, containing 12 independents, four satellites and four faeders in total). This design maintained a constant total male density across all experimental units while experimentally manipulating morph composition, thereby avoiding confounding treatment effects with differences in overall male density.

**Figure 1.**
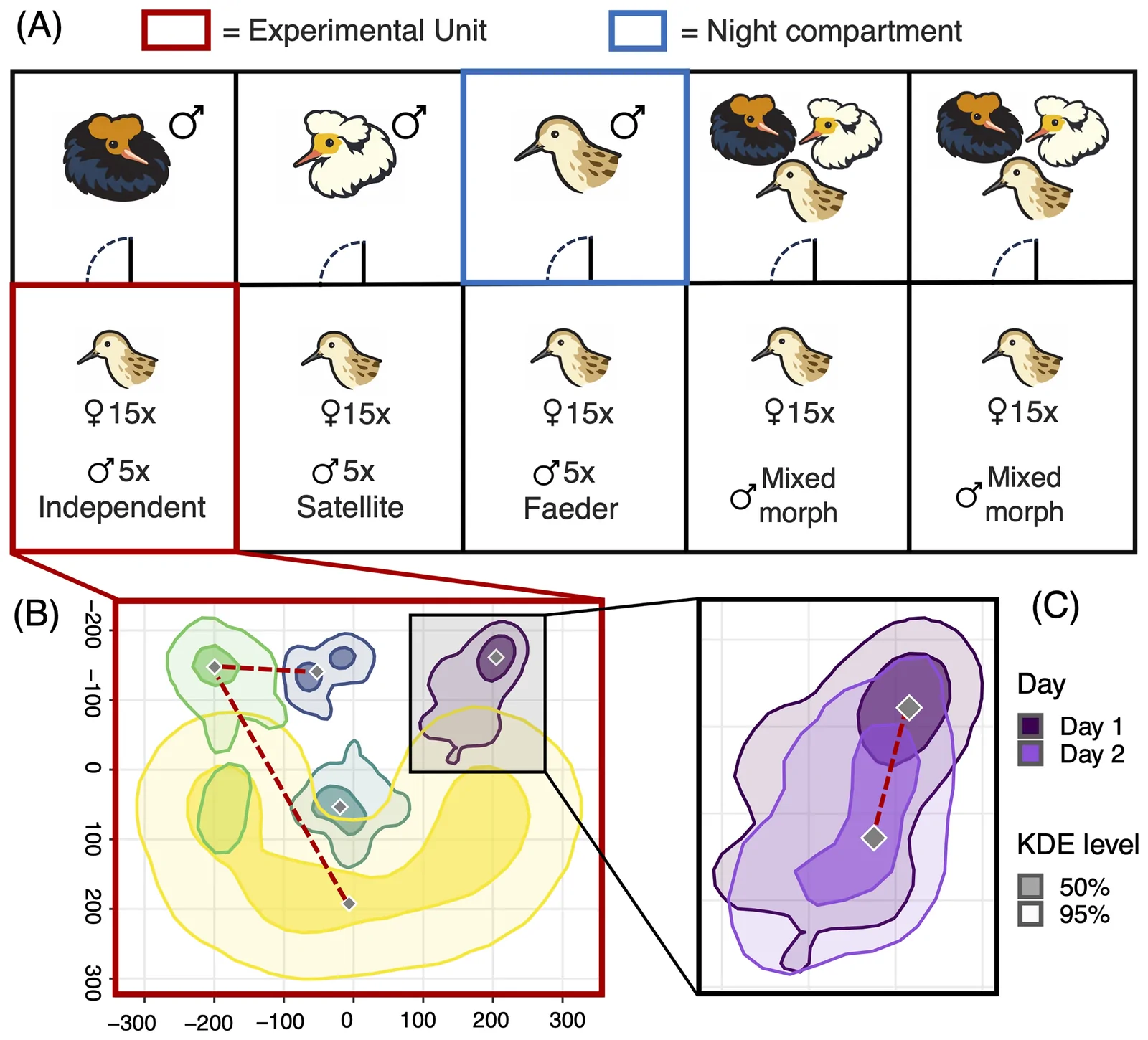
(A) Schematic of the experimental set-up showing the five experimental units with the adjacent night compartments. (B) Example of reconstructed spatial behaviour (kernel density estimates (KDEs)—see main text) of five independents during one experimental session. The two contours correspond to the 95% KDE isopleth (lighter fill) and the 50% KDE isopleth (darker fill). Diamonds indicate centroids of the 50% KDE polygons, and dashed lines provide examples of inter-centroid Euclidean distances. (C) KDEs of the same individual in two consecutive sessions; the dashed line represents the within-individual centroid displacement between sessions.

Leks consisting of five males are commonly observed in nature [52,57,58] and a typical lek has on average only one satellite [52]. The mixed-morph ‘control’ units were used to collect baseline values of space use and compare them to the single-morph units. Owing to limited availability of faeders and satellites, 13 birds took part in the experiment in both years, but were used in different treatment groups (single versus mixed morph). Each unit housed male ruffs of similar ages distribution (average age ± s.d. = 4.24 ± 0.85), and we made sure that females were equally distributed according to their morph.

Experimental sessions began every morning at approximately 06.00, when males were released from the night compartments into the units, and left to freely interact with the females for 3 hours to capture the period of highest lekking activity. Independent males consistently re-established their activity areas in the same locations on consecutive days (see §4), indicating high spatial fidelity consistent with spatial behaviour expected for territoriality. At the end of the session, males were caught and separated from the females until the next morning. All units were equipped with a synchronized video-camera system with a top view on the lek. Videos were recorded at 25 fps.

Experimental males were equipped with plastic tags of different colours, mounted on their backs with a harness for the duration of the experiment. This allowed us to discriminate faeder males from females, as well as to improve the performance of automatic tracking tools (see below). Female movements were not quantified owing to the technical limitations associated with marking and tracking simultaneously such a large number of individuals. To partly account for female spatial behaviour in our analyses, we quantified the spatial distribution of copulations relative to male activity areas (see below), providing an indirect measure of where females interacted with males across treatments. For each experimental session, we analysed the first 45 minutes of video footage. One session every second day was analysed according to a fixed sampling schedule, ensuring full seasonal coverage while limiting data volume. This resulted in a total of approximately 30 experimental sessions per unit-year. Two males (one independent and one satellite) died for unknown reasons during the experiments in 2024 in a control and single-morph unit, respectively, and were not replaced. We repeated the analysis using both the full and truncated datasets (i.e. with and without sessions with missing birds); the results did not vary substantially (see the supplementary material).

### 2.2. Automatic tracking of bird movement

Male movements within units were automatically quantified in the software Loopy (http://loopb.io, Loopbio, GmbH, Austria) via its *Pose detector* function. This function applies a convolutional neural network based on R esNet50 [59], trained in a supervised manner on manually annotated frames, to identify visual features (hereafter ‘keypoints’) within video frames. Each keypoint corresponded to a different individual male, allowing us to track their positions across frames. To achieve this, we selected 10–15 sessions per unit-year and manually annotated a subset of frames to train one separate pose detection model per unit-year. A total of approximately 1000 frames across selected sessions (mean ± s.d. = 1070 ± 185 frames per unit-year) were manually annotated by four coders. After the training phase, each pose detector was then used to automatically track male ruffs across the full dataset (i.e. 30 experimental sessions). The pose detector returned *x* and *y* coordinates for each frame and keypoint (mean frame number per session ± s.d.: 65 744 ± 517). A subset of sessions that could not be successfully tracked using the trained pose detectors (owing to the tags not being visible or falling off the birds during the experiment, or the model crashing for unclear reasons) were annotated manually or discarded. The final sample size was 26.8 ± 3.74 (mean ± s.d.) sessions per unit-year (range 17–30).

After acquiring keypoint coordinates for each experimental session, we subsequently used custom-written software for correcting for camera lens distortion and obtain true Euclidean distances between individuals (see the supplementary material).

### 2.3. Validation of the automated annotation results and data cleaning

We first identified and removed incorrect detections (e.g. female or inanimate objects detected as males, or identity errors). Keypoints outside the perimeter of each unit were deleted, and a maximum speed threshold (4.61 m s^−1^) derived from a subset of *n* = 10 manually annotated sessions, was used to filter detections with unrealistically high velocities (mean ± s.d. data loss per unit-year: 0.32% ± 0.12%).

We then compared a subset of the AI-based annotations (*n* = 5 sessions per unit-year; *n* = 250 annotations per session) to the respective manual annotations, in order to validate the automatic annotation models. The comparison between the automatic and the manual annotations demonstrated consistent agreement across units, supporting the reliability of the automated annotation models (see the supplementary material for details).

### 2.4. Quantification of space use

Space use within the units was quantified from *x* and *y* coordinates using the R packages *amt* [60]. For each daily session, we estimated utilization distributions (UDs) for each bird using kernel density estimates (KDEs), a widely used method in movement ecology that estimates probability density from spatial point data [61] (figure 1B). Two-dimensional KDEs were computed for each male in each session using the *kernelUD* function of the *adehabitatHR* package [62], with the smoothing parameter set to the reference bandwidth (h = ‘href’; see [63]). We estimated UDs using standard KDEs without applying boundary constraints [64]. From each KDE, the 50% and 95% UD contours (i.e. isopleths) were extracted, representing ‘core areas’ and ‘total activity areas’, respectively (figure 1B). All analyses were conducted using projected coordinate data (i.e. planar coordinates in cm). To quantify the area of each bird's UD, we calculated the planar area (in cm^2^) within the 50% and 95% isopleths for each bird and session using the *sf* package [65]. For both KDE contours, we also extracted the geometric centroid using the function *st_centroid*, and the Euclidean distance between centroids was calculated for all possible pairs of males within the same unit (figure 1B). We were also interested in estimating within-individual space-use stability over time for activity areas. To quantify temporal stability of space use, we calculated within-individual centroid ‘displacement’ as the Euclidean distance between centroids of each male between consecutive sessions (figure 1C). Smaller displacement values indicate greater spatial stability, reflecting the repeated use of similar activity areas across time. Together, these metrics provide complementary measures of the size, stability and exclusivity of space use and were used as indirect proxies of territorial behaviour, rather than direct behavioural measures of territory defence.

Last, we investigated the extent to which activity areas of male ruffs overlapped during the experimental sessions, as a proxy of exclusive space use. For all individuals of the same unit, pairwise overlap values were calculated separately for 50% and 95% KDE polygons by computing the spatial intersection between two polygons in cm^2^. Overlap proportions were then obtained by dividing this intersection area by the total area of the focal individual's KDE polygon. As a result, overlap values are asymmetric: the proportion of A overlapping B was not necessarily equal to the proportion of B overlapping A, because each is normalized by a different area.

### 2.5. Blood sampling and hormone assays

In 2024, we collected blood samples from all experimental males (five independents, five satellites and five faeders from the single-morph units, and three independents, one satellite and one faeder from each of the two mixed-morph units) five times during the experiment, starting on day 1, and subsequently at two-week intervals until the end of the experiment. Blood samples were collected immediately following the morning experimental session and prior to returning males to their overnight compartments. We collected approximately 250 µl of blood into heparinized micropipettes at the end of experimental sessions (approx. 09.30), stored it on ice, and centrifuged the samples at 2000 rpm for 9 min; plasma was separated and stored frozen (−20°C) until further analysis. Testosterone concentrations were quantified using a direct radioimmunoassay following the protocol described by Goymann *et al.* [66]. The assay detection limit ranged from 32 to 40 pg ml^−1^, intra-assay variation was 5.0% ± 1.7%, and inter-assay variation was 2.0%. For detailed information on hormone extraction and assays procedures, see the supplementary material.

### 2.6. Spatial distribution of copulations

To investigate whether mating events were spatially associated with male activity areas, and whether this relationship differed among morphs or experimental treatments, we quantified the location of copulations relative to each male's daily UD. We annotated copulations occurring within a standardized 10-minute subsample (5 minutes at the beginning and 5 minutes at the end) of each experimental session, providing a representative estimate of mating activity throughout the session. We included in the copulation count all the events where a female was crouching in the typical solicitation posture, followed by mounting by a male. First, for each of the copulation events, we extracted spatial coordinates and determined whether it occurred inside or outside the 50% and 95% isopleths of the copulating bird's daily KDE, using the function *st_intersects* from the *sf* package [65]. Second, to quantify the spatial dispersion of copulation events, we calculated the area encompassed by copulation locations for each male bird within each session. When at least three copulations per male were available, we constructed a convex hull polygon representing the minimum convex area (in cm^2^) enclosing all events.

## 3. Statistical analyses

We tested our hypotheses using a series of mixed-effect models that included bird ID and unit-year fitted as random intercepts, to account for repeated measures. Unless otherwise specified, full models fit significantly better than the respective null models lacking test predictors or interactions. Details on model structure, diagnostics and implementation are provided in the supplementary material.

### 3.1. Core and total activity area

We tested whether experimental treatment (single vesus mixed morph) affected core and total activity areas (50% and 95% KDEs) across morphs using linear mixed-effect models with Gaussian error distribution and log-transformed KDE areas as response variables. Fixed effects included morph, treatment, their interaction, year and age. The interaction term specifically tested whether treatment affected male morphs differentially. To assess seasonal changes, we modelled centred ordinal day and its interaction with morph and treatment, allowing morph-specific seasonal trajectories to vary between treatments. Linear and quadratic trends were compared among morph–treatment combinations using estimated marginal trends.

### 3.2. Pairwise centroid distances

Pairwise Euclidean distances between centroids (50% and 95% KDEs) were analysed using multilevel models with a lognormal error distribution. Dyad type (morph combination and treatment, for example, satellite–satellite (single), satellite–independent (mixed) etc.) was the main predictor. Random effects accounted for repeated measures of individuals (multi-membership), dyads and unit-year. Pairwise comparisons among dyad types were obtained from estimated marginal means. Within-individual spatial stability was analysed as centroid distances between consecutive sessions (figure 1C), with treatment, morph, their interaction, year and age as fixed effects.

### 3.3. Overlap proportions

Overlap proportions (50% and 95% KDEs) were modelled using beta mixed models with morph, treatment and their interaction as predictors, including age as a covariate and partner ID as additional random intercepts. A zero-inflation component was included for 50% KDEs.

### 3.4. Testosterone levels

Log-transformed testosterone concentrations were modelled as a function of morph and age (linear and quadratic terms). As the morph : age interaction was not significant, the reduced model was retained. We then tested whether testosterone levels were associated with space-use metrics (areas, centroid distances and overlap) including interactions with morph.

### 3.5. Spatial distribution of copulations

The probability that copulations occurred within 50% and 95% KDEs was analysed using binomial mixed models with morph, treatment, their interaction and KDE area as fixed effects. Differences in spatial dispersion among morphs were assessed using convex hull areas as dependent variable in linear mixed models.

## 4. Results

### 4.1. Core and total activity areas

Core areas (50% KDE) differed among morphs and depended on the experimental treatment (interaction morph x treatment: *F*_2_ = 7.46, *p* = 0.008). Independents occupied the smallest core areas, particularly in the single-morph treatment, whereas faeders and satellites used larger areas and did not differ from one another (figure 2A). In the mixed-morph units, morph differences were less pronounced, with only independents and satellites differing significantly (supplementary material, table S3). Within morphs, comparisons between experimental treatments showed that independents had smaller areas in the single-morph compared to the mixed-morph treatment (50% KDE: estimate = −0.815, s.e. = 0.362, *p* = 0.035), while satellites and faeders did not differ between treatments (figure 2A; supplementary material, table S3). Thus, the interaction term was mainly driven by area differences in the independent morph between treatments.

**Figure 2.**
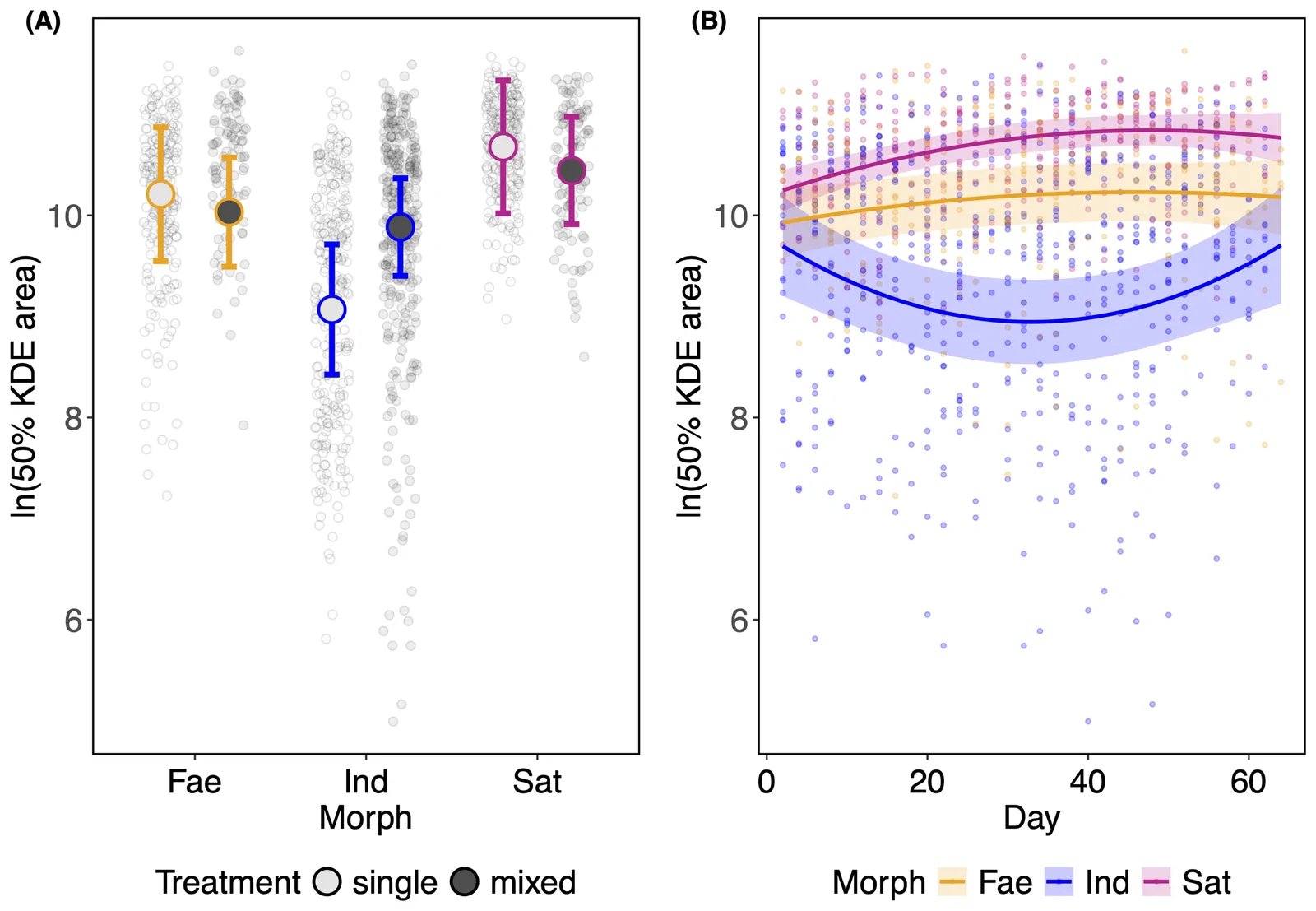
Core areas (50% KDE) differ in independent males across treatments and show nonlinear temporal patterns over the lekking season. (A) Morph variation in core area (i.e. 50% KDE) measured in single- and mixed-morph units. Points and error bars represent estimated marginal means (± 95% confidence interval (CI)) from a linear mixed model fitted on log-transformed core areas. (B) Seasonal patterns in core areas for each morph in the single-morph treatment. Lines and shaded ribbons show fixed-effect predictions ± 95% CI from morph-specific linear mixed-effect models fitted to log-transformed core areas.

For seasonal trends, the model including the full three-way interaction between day, morph and treatment fitted significantly better than the reduced model ($\chi_{12}^{2}$ = 39.14, *p* < 0.001). However, the three-way interaction terms were estimated with some uncertainty (supplementary material, table S5). To facilitate biological interpretation, we therefore analysed each morph separately. Independent males showed a pronounced seasonal contraction of their core areas, which became smallest around the middle of the breeding season before expanding again towards its end (figure 2B). This pattern was similar in both treatments, as there was no evidence for a treatment × season interaction ($\chi_{2}^{2}$ = 3.23, *p* = 0.199). The reduced model lacking the interaction term showed a significant quadratic seasonal pattern (*β* = 0.0025 ± 0.0006, *p* < 0.001; figure 2B). In satellites, core areas increased linearly over the season only in the single-morph treatment (*β* = 0.013 ± 0.003, *p* < 0.001), indicating treatment-dependent seasonal dynamics (supplementary material, table S5). Faeder males showed no detectable seasonal changes in core area size, and seasonal patterns did not differ between treatments ($\chi_{2}^{2}$ = 1.96, *p* = 0.375). Models for total activity areas (95% KDE) showed similar results (supplementary material, tables S4 and S6).

### 4.2. Pairwise centroid distances

The spatial segregation between males differed significantly among dyad types for both 50% and 95% KDEs (supplementary material, figure S4A). Faeder–faeder and satellite–satellite dyads were consistently the most spatially cohesive, maintaining the smallest centroid distances. Satellite–satellite dyads did not differ credibly from faeder–faeder dyads (95% credible intervals overlapping zero). By contrast, independent–independent dyads were the most spatially separated. In particular, independent dyads in the single-morph treatment showed the greatest centroid distances overall, with an estimated increase of approximately 0.7 log-units (95% credible intervals: 0.33–1.1), corresponding to approximately a twofold increase in spatial distance relative to faeder–faeder dyads (supplementary material, tables S7 and S8). Independent dyads in mixed-morph groups did not differ credibly from those in single-morph groups, although mean centroid distances were consistently higher in the latter. Centroid distances among mixed-morph dyads (faeder–independent, faeder–satellite, independent–satellite) largely overlapped, indicating broadly similar levels of spatial distancing among these categories (supplementary material, figure S4A).

With regard to space-use stability, that is, within-individual centroid displacement between sessions, we found a significant interaction between treatment and morph ($\chi_{2}^{2}$ = 7.68, *p* = 0.024). Independents showed significantly smaller centroid distances in the single-morph treatment compared to the mixed-morph (estimate = −0.429 ± 0.151 s.e., *p* = 0.009), suggesting higher space-use stability across sessions, while satellites and faeders did not differ between treatments in space-use stability (supplementary material, figure S4B). Independents also showed smaller centroid displacement values than satellites in the single-morph units (supplementary material, table S9).

### 4.3. Spatial overlap

Morphs differed in the degree to which their core activity areas overlapped, with independent males showing the strongest spatial segregation, particularly in single-morph leks. The beta model for 50% KDE overlap proportions revealed a significant interaction term in the zero component, indicating that the probability of zero overlap varied strongly with morph and treatment (figure 3A). Independents had a significantly higher probability of complete segregation (zero overlap) than the other morphs, and this effect was amplified in the ‘single-morph’ treatment (interaction treatment x morph: *β* = 2.45 ± 0.17, *z* = 14.22, *p* < 0.001). By contrast, both faeders and satellites showed a lower probability of zero overlap in the single-morph compared to the mixed-morph group. Conditional overlap magnitude (i.e. given overlap greater than 0) showed only weak effects of treatment and its interaction with morph (supplementary material, table S10). Thus, differences among morphs in core-area overlap were driven primarily by whether males shared any portion of their core areas at all, rather than by differences in the amount of overlap when overlap occurred. The beta model for the 95% KDE contour produced similar results, with independents in the single-morph treatment showing less overlap than in the mixed-morph treatment (Δ = 0.772 ± 0.328, *p* = 0.019), whereas overlap values for satellite and faeders did not differ between the two treatment groups (figure 3B; supplementary material, table S11). Residual diagnostics for this latter model, however, indicated significant under-dispersion (DHARMa test: dispersion = 0.83, *p* < 0.001).

**Figure 3.**
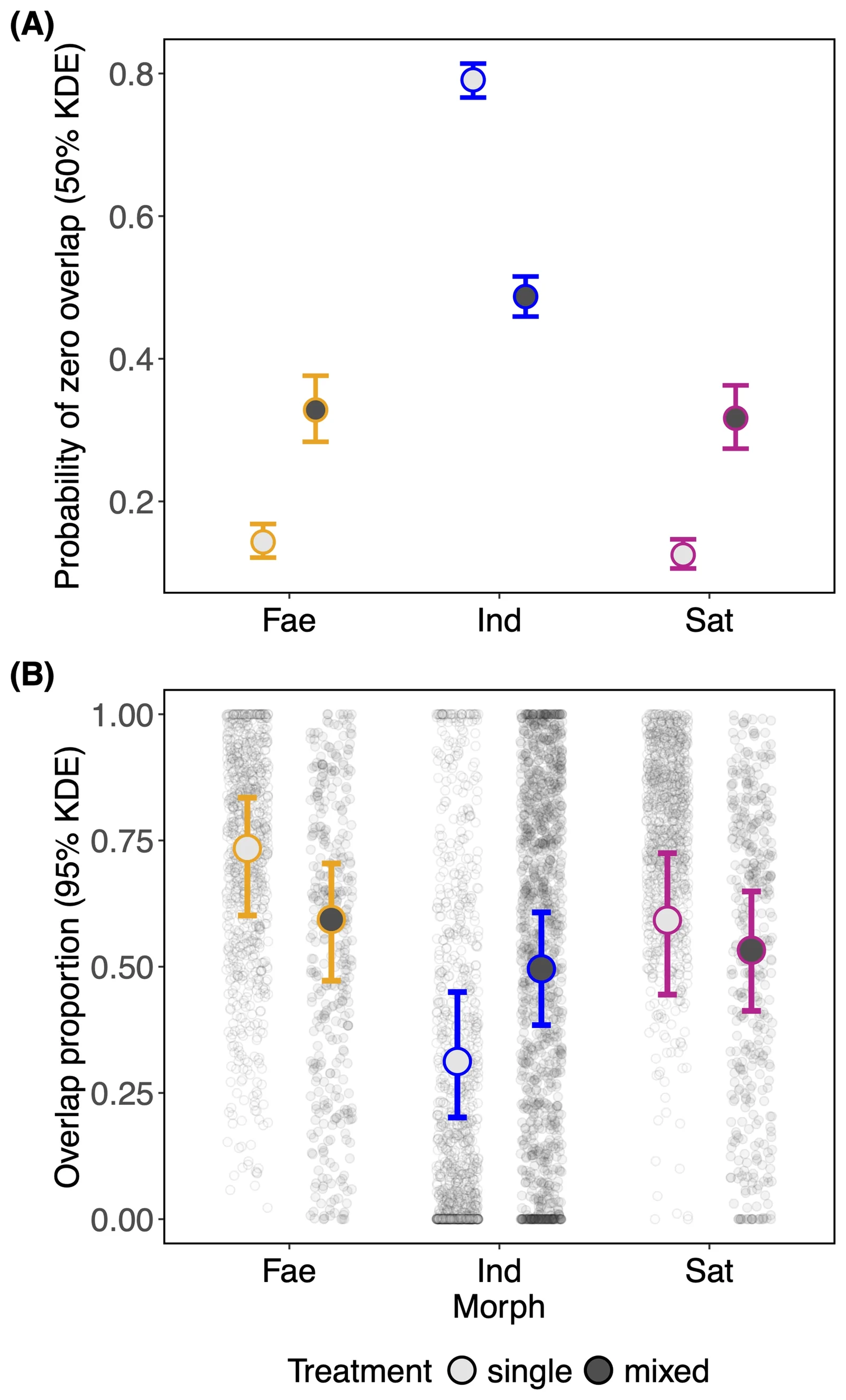
Spatial overlap between ruffs was lower for independents than faeders and satellites, with overlap being the smallest in single-morph treatment. (A) Predicted probabilities of zero overlap (50% KDE) from the zero-inflation component of the beta model. Points represent estimated marginal means and bars the ±95% confidence intervals (CIs). (B) Distribution of overlap proportions for the 95% KDEs across morphs and treatments. Points indicate model-estimated marginal means (±95% CIs).

### 4.4. Testosterone levels

There was no evidence that the relationship between age and testosterone differed among morphs (*F*_4,109_ = 1.48, *p* = 0.21), therefore the interaction terms were excluded from the final model. Independents had higher testosterone levels than faeders (*β* = 0.96 ± 0.23 s.e., *t* = 4.20, *p* < 0.001) and satellites (*β* = 1.05 ± 0.22 s.e., *t* = 4.74, *p* < 0.001), whereas satellites did not differ from faeders (*β* = −0.09 ± 0.27 s.e., *t* = −0.34, *p* = 0.73; figure 4). Age showed a significant quadratic relationship with testosterone, with testosterone peaking at intermediate ages (*β* = −0.024 ± 0.010 s.e., *p* = 0.022).

**Figure 4.**
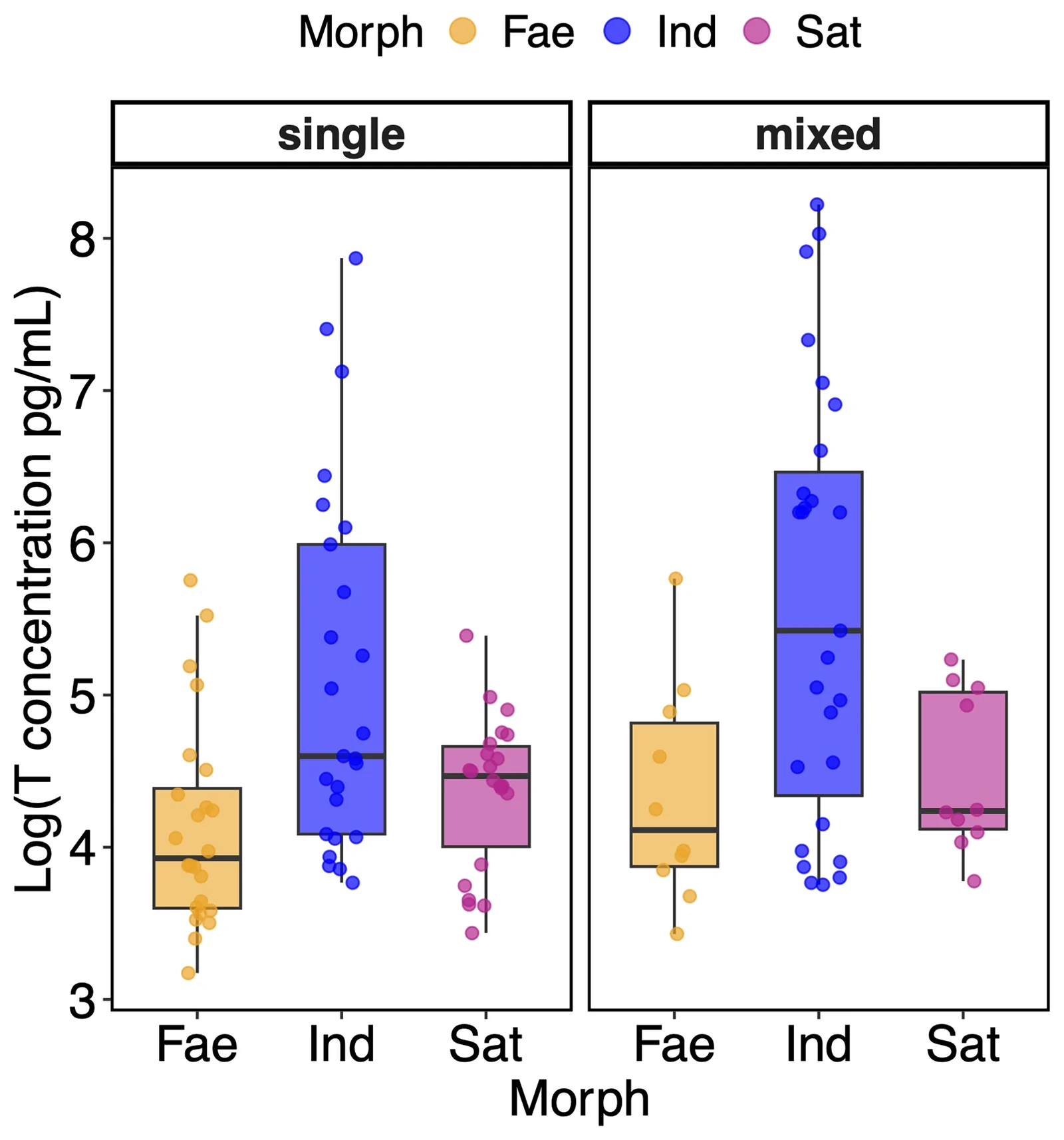
Log-transformed testosterone concentrations (pg ml^−1^) of ruff males sampled in 2024 are higher in territorial independents than non-territorial satellites or faeders. Each male was sampled five times during the lekking season. Different panels show males in different treatment groups (single versus mixed morph).

In the three models for KDE area (50% KDE), centroid distance and overlap, the interaction term between testosterone levels and morph did not improve model fit compared to all the respective null models (area: $\chi_{2}^{2}$ = 4.88, *p* = 0.08; overlap: $\chi_{2}^{2}$ = 5.941, *p* = 0.051; inter-centroid distance: $\chi_{2}^{2}$ = 2.59, *p* = 0.273). Furthermore, testosterone levels as an independent predictor (i.e. in the reduced model without the interaction with morph) did not co-vary with area, overlap and centroid distance values in any of the models (supplementary material, table S12). The same results were found in all models for the 95% KDEs (supplementary material, table S13). Thus, within-season fluctuations in neither of those measures co-varied with testosterone variation within or between morphs.

### 4.5. Spatial distribution of copulations

We observed a total *n* = 565 copulation events in both experimental seasons, distributed unevenly across morphs (supplementary material, figure S5). Regarding the position of copulation events relative to core areas (50% KDEs), the full model did not differ significantly from the null model without the morph x treatment term ($\chi_{2}^{2}$ = 1.019, *p* = 0.601). Only five copulations occurred outside of the 95% KDE polygon (*n* = 1 by a satellite; *n* = 4 by independents); therefore, no further analyses were conducted for total activity areas. The size of copulation areas (convex hulls) increased strongly with the number of copulations used to construct the polygon (*β* = 0.425 ± 0.098, *p* < 0.001). After controlling for this effect, polygon area differed modestly among morphs (*F* = 5.55, *p* = 0.048), with satellites exhibiting larger polygons than independents (estimate = 1.592 ± 0.515, *p* = 0.034), whereas faeders did not differ significantly from either morph.

## 5. Discussion

High resolution tracking of small-scale movements in male ruffs revealed consistent morph-specific space-use patterns both in control and in experimentally manipulated leks.

Independent males occupied overall smaller activity areas compared to the two floater morphs, faeders and satellites, and exhibited clear spatial segregation consistent with territorial behaviour. Activity areas and inter-centroid distances in independents closely resembled those reported in wild ruffs [51], indicating that ruffs exhibited natural lekking behaviour in our captive set-up. Independents also showed the smallest within-individual centroid displacement across sessions, suggesting that territories were spatially stable over time [67]. Furthermore, independents in single-morph units clearly differed from independents in mixed-morph units, which showed larger core areas and reduced spatial segregation. These differences have two non-mutually exclusive explanations. First, differences in lek size (five versus three independents in single and mixed-morph units, respectively) may affect spatial dynamics in independents via increased competition for space, constraining male territory size independently of their resource holding potentials and competitive abilities when more independents lek together. For example, Hogan-Warburg [51] reported that in larger leks, male ‘residencies’ (identified then as circular patches of trampled grass on a lek) are typically approximately 1 m apart, as opposed to a distance of approximately 1.5 m in smaller leks in the wild. Second, in mixed-morph leks, independents had to both defend courts and manage interactions with satellite males, namely attempting to monopolize opportunities for co-displays. Satellites constitute an important social resource on leks, as co-display diverts female visits and copulations to the court of co-displaying male pairs [4,48,49]. Thus, independent males in mixed-morph leks faced behavioural demands associated with satellite interactions that were absent from the single-morph treatment. These additional behavioural demands on mixed leks may disrupt regular spacing and lead to larger or less spatially stable activity areas. However, because the mixed-morph treatment also contained fewer independent males than the single-morph treatment, the effects of floater presence and reduced independent density cannot be fully disentangled in the present experiment. We intentionally maintained a constant total male density across treatments to avoid introducing an additional confounding effect of overall male density on spatial behaviour, but future experiments employing a combined manipulation of morph composition and male density could clarify their relative role in shaping space use.

In addition, we observed seasonal variation in space use in independents, with larger core areas at the beginning and end of the breeding season than mid-season. This pattern indicates that space use changed over time and coincided with the period of higher observed copulation rates (supplementary material, figure S5). One possible explanation is that males reduced the size of their activity areas during periods of increased mating activity, potentially reflecting a greater investment in territorial defence when reproductive opportunities were the highest. As lek attendance is a key determinant of mating success in ruffs [4,52,68], males may benefit from increasing territorial defence and spatial stability when mating opportunities peak. While copulation distribution did not differ between treatments in independents, seasonal fluctuations in core areas probably reflect behavioural adjustments to temporal variation in reproductive competition, although whether this reflects changes in female receptivity, male behaviour or other seasonal factors cannot be determined from the present study. Overall, our results indicate that space use in independents appears primarily shaped by competitive dynamics on the lek, with flexibility driven by lek size (number of competitors) and seasonal fluctuations in reproductive opportunities.

Despite clear morph- and age-related differences in circulating testosterone, we found no evidence that within- or between-individual variation in spatial metrics covaried with circulating testosterone levels. Independents showed considerable variation in testosterone concentrations, and males peaked at different time points during the breeding season (supplementary material, figure S6), yet spatial behaviour did not appear to be associated with measured endocrine variation. Thus, short-term changes in testosterone levels do not seem to mediate fine-scale spatial organization in ruffs. This finding is noteworthy because it suggests that, despite pronounced morph-specific endocrine differences, short-term fluctuations in circulating testosterone are unlikely to explain within-season variation in spatial behaviour, although this interpretation should be treated with caution given the limited sample size of the endocrine dataset. While testosterone is known to regulate aggression and territoriality in birds and other vertebrates [25,27,35], its role in within-individual, short-term behavioural variation remains contentious. For example, golden-collared manakins (*Manacus vitellinus*) [69] and lance-tailed manakins (*Chiroxiphia lanceolata*) [31] maintain elevated courtship rates in spite of a decrease in testosterone levels during the breeding season. Possibly, a critical threshold of androgens early in the season is necessary to activate territory establishment and aggressive/display behaviour, whereas sustained elevated levels are not required for their maintenance later in the season [31,69]. Thus, testosterone may function primarily as an initial activator rather than a continuous modulator. Furthermore, satellites and faeders in both treatment groups consistently maintained lower testosterone concentrations than independents (figure 4). This pattern aligns with previous work on endocrine regulation in ruffs, demonstrating morph-specific hormonal profiles linked to the inversion haplotypes underlying ARTs [46,53]. In particular, differential expression of *HSD17B2*, a gene within the supergene, alters androgen metabolism by increasing the conversion of testosterone to androstenedione in the blood of satellite and faeder males but not of independents, thereby maintaining lower circulating testosterone levels in the two non-territorial morphs [53]. Together, these findings suggest that although testosterone differs consistently among genetically determined morphs, short-term endocrine fluctuations are unlikely to be the primary mechanism underlying within-season variation in spatial behaviour. These genetically mediated endocrine differences probably impose physiological constraints on hormonal flexibility in ruffs, resulting in limited plasticity in testosterone regulation.

Interestingly, both floater morphs also did not adjust their spatial behaviour in experimentally manipulated leks. In single-morph units, satellites and faeders did not occupy vacant social niches nor exhibit spatial signatures consistent with (incipient) territoriality. Specifically, we observed no reduction in core or total activity areas in single-morph compared to mixed-morph units—which would indicate territory establishment or consolidation over time—nor did we detect increased inter-individual segregation. The distribution of copulations also did not differ between the two treatment groups. By contrast, both satellites and faeders were spatially *closer* to other males in single-morph than in mixed-morph leks, strongly suggesting that both floater morphs formed cohesive spatial arrangements. In satellites, spatial cohesion probably resulted from cooperative (i.e. co-display) behaviour, as they frequently co-displayed with one another, occasionally forming small display groups of up to three individuals (G.S. Spezie, C.K. Küpper 2024, unpublished data). Indeed, satellites are obligate cooperators that engage in joint displays with independent males on natural leks [58]. Conversely, patterns of spatial cohesion in faeders were somewhat more unexpected. Unlike satellites, faeders are non-cooperative sneaker males that mimic females in appearance and behaviour [44]. In natural leks, their reproductive tactic therefore relies on sneaking matings opportunistically. The spatial cohesion observed here may therefore reflect a general tendency of faeders to remain close to other males, regardless of morph, in order to maximize mating opportunities whenever they arise. Furthermore, satellites showed low space-use stability, suggesting dynamic, coordinated movement, whereas faeders showed intermediate values between satellites and independents, and were more consistently clustered together in the same areas, potentially where females aggregated. Additional information on space use in females would help determine if faeders mirror female spatial behaviour or exhibit a distinct spatial pattern. Overall, these patterns of social cohesion in both floater morphs suggest that reduced androgen levels probably facilitate social tolerance and spatial aggregation, important for cooperative or sneaker tactics that depend on proximity to other males. Conversely, higher testosterone concentrations in independents may promote spatial segregation and territorial defence. Interestingly, in wired-tailed manakins (*Pipra filicauda*), resident territory owners with low testosterone levels were shown to attract and maintain stable cooperative partnerships with more subordinate floaters at their display arenas [26], underscoring how low androgen levels can mediate gregarious or social behaviours both in floaters and in resident males.

Overall, the absence of hormonal and behavioural shifts in floater morphs argues against rapid, reversible tactic switching. Satellites and faeders did not adopt—either rapidly or gradually—space-use patterns consistent with territoriality, indicating that the spatial component of floater strategies are highly canalized in both morphs, leaving little scope for behavioural plasticity under the social conditions tested. In contrast to other species with genetically determined ARTs, for example, swordtail fish *Xiphophorus nigrensis*, where sneakers can switch between sneaking and courtship behaviour when larger ‘courter’ males were experimentally removed [70,71], our results suggest that ruff morphs represent fixed specializations shaped by strong genetic and physiological constraints. Thus, although selection is generally expected to favour endocrine mechanisms that enable behavioural plasticity in response to social opportunities [72,73], the costs and constraints associated with the ruff morphs may instead promote more specialized strategies that perform optimally under low-density social conditions. Nonetheless, some degree of conditional variation may still occur *within* morphs. Among independent males, for example, previous work has distinguished between territorial ‘resident’ and non-territorial ‘marginal’ males. Residents typically defend display courts and exhibit high site fidelity during the breeding season, whereas marginal males—often younger or lower-condition individuals—fail to secure territories and instead adopt a more mobile, floater-like strategy [49,50,52]. These patterns suggest that conditional tactics may be nested within an otherwise genetically determined ART. The substantial variation in spatial behaviour observed among independents in our study (figures 2 and 3) is consistent with this scenario. Because our experimental population included both 1-year-old and older independent males, some of this variation could reflect age-related differences in the expression of resident and marginal tactics. However, age was included as a predictor in our statistical models and did not explain a significant proportion of the observed variation. Our experimental design may nevertheless have limited our ability to detect fully expressed marginal behaviour. Future experimental studies allowing movement *between* display arenas may therefore be important for capturing the transition between marginal and resident tactics in independent males, and allowing inter-lek movement would probably improve our understanding of spatial behaviour (and plasticity) in floaters. Finally, although floater morphs may not establish territories, they may still flexibly adjust other morph-specific behaviours in response to variable social environments. For instance, floaters may benefit from context-dependent modulation of aggressive behaviour despite lacking the ability to establish and defend stable territories.

More broadly, our findings contribute to the growing recognition that genetic determination of an ART may not necessarily entail complete behavioural inflexibility, but rather that the degree and form of plasticity may vary among components of a tactic and across taxa. In species with genetically determined ARTs, social context can influence the expression of alternative behaviours, as demonstrated by tactic switching in some fishes [70,71], whereas our findings show that spatial behaviour in breeding males is socially structured but largely canalized, highlighting the value of experimental manipulation of the social environment. Understanding which components remain plastic, and which are developmentally or physiologically canalized, may therefore be key to explaining how genetically determined ARTs are maintained despite variation in social environments. Future work should examine behavioural plasticity beyond space use, particularly by incorporating measures of aggression and courtship behaviour, to better understand how genetic, endocrine and social factors interact in shaping ARTs.

## Supplementary Material



## Data Availability

Datasets and codes can be accessed as the electronic supplementary material [74].
