# Supplementary material for: Distinct space-use patterns are associated with alternative reproductive tactics in lekking ruffs Calidris pugnax
Source: R Soc Open Sci. 2026 Nov 11;13(11):262020. doi: 10.1098/rsos.262020 (PMC13750937; doi:10.1098/rsos.262020)
Supplement: Supplementary file 1 [file rsos.262020.zip › rsos.262020/rsos262020_si_001.docx]

**Distinct space use patterns are associated with alternative reproductive tactics in the ruff *Calidris pugnax***

**Electronic Supplementary Materials**

1. **Lens distortion correction**

The top-view camera recordings were strongly distorted, therefore, to obtain true Euclidean distances between individuals, image coordinates had to be mapped from the raw image plane to an undistorted planar Euclidean coordinate system representing the flat ground surface. This mapping required both intrinsic and extrinsic camera parameters, because it had to account for lens distortion as well as the position and orientation of the camera relative to the observed plane (Hartley et al. 2004). The intrinsic parameters were estimated by camera calibration following Zhang’s method (Zhang 2000), using images of a chessboard pattern with known geometry. The extrinsic parameters were determined from the measured height of the camera above the ground and the known dimensions of the experimental unit. Together, these parameters defined the image-to-ground transformation matrix, which allowed each pixel location to be assigned to a corresponding position in the planar Euclidean reference frame. Figure S1 illustrates how image pixels are mapped into a Euclidean coordinate system. In particular, structures that appear curved in the original image are represented as straight in the transformed view, consistent with their true geometry. Bird positions, represented by their centroids in image coordinates, were then projected into this ground-plane coordinate system, and inter-individual distances were calculated as Euclidean distances between centroid positions in the transformed space. This procedure corrected for both perspective effects and lens distortion, thereby enabling distance measurements in biologically meaningful metric units.

Hartley R, Zisserman A. Multiple View Geometry in Computer Vision. 2nd ed. Cambridge University Press; 2004.

Zhang Z. A flexible new technique for camera calibration. IEEE Transactions on Pattern Analysis and Machine Intelligence. 2000;22(11):1330–1334.

Figure S1 An original image (top) and its corresponding undistorted version (bottom). A mesh of 50 × 50 cm grids back-projected onto the lek, while accounting for image distortion, is illustrated in green. The applied transformation allows the Euclidean distance between the two projected bird positions to be calculated directly, as indicated in the undistorted image.

2. **Validation of automated detection and identification**

**Methods**

To assess the performance of the automated detection pipeline, we compared model predictions against manual annotations on a subsampled validation dataset. For each experimental unit, we randomly selected a subset of videos and extracted one frame every 250 frames to reduce temporal autocorrelation and ensure independence among sampled observations. All selected frames were manually annotated by four human scorers, excluding those used during model training. For each annotated frame, all visible individuals were labeled and assigned unique identities. The automated predictions were then matched to the corresponding manual annotations based on individual identity. Validation analyses were conducted separately for each experimental unit, as independent models were trained per unit (see main text).

*Detection performance*

We first evaluated the ability of the model to correctly detect individuals. For each individual in each frame, we classified detections as true positives (TP; individual present in both manual and automated annotations), false negatives (FN; individual present in manual annotations but not detected automatically), or false positives (FP; individual detected automatically but not present in manual annotations). Based on these classifications, we calculated *precision* (TP / [TP + FP]) and *recall* (TP / [TP + FN]) for each experimental unit.

*Spatial accuracy*

To quantify the accuracy of the estimated positions, we calculated the Euclidean distance (in cm) between manually annotated and automatically predicted coordinates for each individual in each frame. We summarized spatial error distributions using mean, median, and standard deviation, and visualized their distributions for each model. In addition, we calculated the proportion of detections falling within predefined spatial thresholds (5cm, 10cm, 15cm, 20cm) of the manual annotations to provide an intuitive measure of localization accuracy.

*Identity assignment*

Because automated detections were associated with individual identities, we further assessed the consistency of identity assignment at the frame level. As a proxy for identity errors, we identified cases where the spatial discrepancy between manual and automated annotations exceeded a predefined threshold of 20 cm, which likely indicates incorrect identity assignment or severe localization error. We report the proportion of such cases for each experimental unit.

*Comparison with human annotation variability*

To contextualize model performance relative to annotation uncertainty, we quantified human inter-observer variability using a subset of validation data from 2024. For these frames, two independent human annotators provided manual annotations for the same individuals. We calculated the Euclidean distance (in cm) between the coordinates provided by the two annotators for each individual in each frame. These distances were summarized using the same metrics as for the model validation (mean and median error) and used as a baseline measure of human annotation variability. To facilitate comparison between automated and human performance, we additionally calculated the ratio between model error and human–human disagreement for each experimental unit. Values close to 1 indicate model performance comparable to human annotators, whereas higher values indicate reduced model precision relative to human agreement.

*Scope of validation*

Because validation frames were sampled at fixed intervals (every 250 frames), analyses were restricted to frame-wise detection, identification, and localization performance. This approach does not capture temporal tracking errors such as track fragmentation but provides a robust estimate of model performance at independent time points.

**Results**

*Detection performance*

Across all experimental units and both years, the automated system showed consistently high detection performance. Precision was equal to 1 in all cases, indicating the absence of false positive detections. Recall values were also high, ranging from 0.882 to 0.973 across units and years, demonstrating that the majority of individuals present in the manually annotated frames were successfully detected by the model. Recall was generally slightly higher in 2024 compared to 2023, with the highest value observed in the unit 5 in 2024 (0.973). Table S1 provides a summary of these statistics.

*Spatial accuracy*

Spatial accuracy of the automated annotations was high across all experimental units. Mean Euclidean error ranged between 5.01 and 7.80 cm in 2023 and between 3.05 and 6.47 cm in 2024 (Table S1). The proportion of detections falling within predefined spatial thresholds further illustrates model performance (Table S2). At a strict threshold of 5 cm, performance varied substantially across experimental units, ranging from 0.585 to 0.951 in 2023 and from 0.653 to 0.949 in 2024. Given that the birds are approximately 20 cm in body length, differences in annotation may arise from slight inconsistencies in the exact point assigned on the bird’s body rather than true localization error. Indeed, increasing the threshold to 10 cm resulted in consistently high accuracy across all units (≥0.940 in 2023 and ≥0.977 in 2024). At 15 cm and 20 cm thresholds, nearly all detections were within the specified range (>0.987 in all cases). Performance was generally improved in 2024, with unit 5 showing the highest spatial accuracy (mean error = 3.05 cm; 99.6% of detections within 5 cm). Despite overall high accuracy, standard deviations were relatively large (up to 34.1 cm), reflecting the presence of occasional large errors.

*Identity assignment*

The rate of potential identity errors, defined as detections with spatial discrepancies greater than 20 cm, was low across all EUs and years. In 2023, error rates ranged from 0.004 to 0.012, and in 2024 from 0.004 to 0.011 (Table S1). These results indicate that severe mismatches between manual and automated annotations were rare.

*Comparison with human annotation variability*

Inter-observer variability, quantified as the Euclidean distance between annotations from two independent human observers, varied across experimental units (mean error range: 1.48–4.36 cm) (Table S1). In most cases, automated annotation error exceeded human–human disagreement. The ratio between model error and human error ranged from 1.07 to 3.86, indicating that the model was generally less precise than human annotators. The largest discrepancy was observed in unit 3 (error ratio = 3.86), followed by unit 2 (3.34), suggesting reduced model performance in these units relative to human agreement. However, in unit 4, model performance closely matched human-level variability (mean error = 4.35 cm vs. 4.36 cm for humans; ratio ≈ 1), indicating near-human accuracy. Similarly, in unit 5, model performance was only slightly worse than human agreement (ratio = 1.07).

*Summary*

Overall, the automated system achieved high detection accuracy and low spatial error across all experimental units. While performance varied among units, results from 2024 indicate that, in some cases, model accuracy approached human-level annotation variability. The error distributions were strongly right-skewed, with most detections exhibiting small spatial deviations and a small number of large errors.

# Table S1 Summary of the validation statistics for detection performance and spatial accuracy, for all years and experimental units.

| Year | Unit id | Mean error (cm) | Median error (cm) | SD error (cm) | N | TP | FN | FP | Precision | Recall | ID error rate | Mean error human (cm) | Median error human (cm) | Error ratio |
| --- | --- | --- | --- | --- | --- | --- | --- | --- | --- | --- | --- | --- | --- | --- |
| 2023 | Unit 1 | 5.01 | 2.41 | 27.0 | 5599 | 4941 | 658 | 0 | 1.0 | 0.882 | 0.0105 | NA | NA | NA |
| 2023 | Unit 1 | 7.8 | 4.04 | 34.1 | 7141 | 6789 | 352 | 0 | 1.0 | 0.951 | 0.0124 | NA | NA | NA |
| 2023 | Unit 3 | 6.51 | 3.94 | 27.3 | 7305 | 6843 | 462 | 0 | 1.0 | 0.937 | 0.00833 | NA | NA | NA |
| 2023 | Unit 4 | 6.03 | 4.34 | 20.7 | 6981 | 6660 | 321 | 0 | 1.0 | 0.954 | 0.00405 | NA | NA | NA |
| 2023 | Unit 5 | 6.63 | 3.44 | 30.1 | 7286 | 6668 | 618 | 0 | 1.0 | 0.915 | 0.0118 | NA | NA | NA |
| 2024 | Unit 1 | 4.28 | 2.39 | 20.2 | 6215 | 5806 | 409 | 0 | 1.0 | 0.934 | 0.00706 | 3.03 | 2.68 | 1.41 |
| 2024 | Unit 2 | 6.47 | 4.15 | 24.7 | 6824 | 6305 | 519 | 0 | 1.0 | 0.924 | 0.00952 | 1.93 | 1.82 | 3.34 |
| 2024 | Unit 3 | 5.73 | 2.2 | 33.5 | 5710 | 5292 | 418 | 0 | 1.0 | 0.927 | 0.011 | 1.48 | 1.0 | 3.86 |
| 2024 | Unit 4 | 4.35 | 2.9 | 17.6 | 7096 | 6783 | 313 | 0 | 1.0 | 0.956 | 0.00487 | 4.36 | 3.99 | 0.997 |
| 2024 | Unit 5 | 3.05 | 1.81 | 16.0 | 7275 | 7079 | 196 | 0 | 1.0 | 0.973 | 0.00367 | 2.85 | 2.82 | 1.07 |

# Table S2 Proportion of automated detections within predefined spatial error thresholds (5, 10, 15, and 20cm) relative to manual annotations, reported separately for each experimental unit and year.

| Year | UNIT _id | Threshold (cm) | Proportion within threshold |
| --- | --- | --- | --- |
| 2023 | Unit 1 | 5 | 0.951 |
| 2023 | Unit 1 | 10 | 0.989 |
| 2023 | Unit 1 | 15 | 0.989 |
| 2023 | Unit 1 | 20 | 0.989 |
| 2023 | Unit 2 | 5 | 0.675 |
| 2023 | Unit 2 | 10 | 0.976 |
| 2023 | Unit 2 | 15 | 0.987 |
| 2023 | Unit 2 | 20 | 0.988 |
| 2023 | Unit 3 | 5 | 0.675 |
| 2023 | Unit 3 | 10 | 0.966 |
| 2023 | Unit 3 | 15 | 0.991 |
| 2023 | Unit 3 | 20 | 0.992 |
| 2023 | Unit 4 | 5 | 0.585 |
| 2023 | Unit 4 | 10 | 0.94 |
| 2023 | Unit 4 | 15 | 0.994 |
| 2023 | Unit 4 | 20 | 0.996 |
| 2023 | Unit 5 | 5 | 0.735 |
| 2023 | Unit 5 | 10 | 0.979 |
| 2023 | Unit 5 | 15 | 0.987 |
| 2023 | Unit 5 | 20 | 0.988 |
| 2024 | Unit 1 | 5 | 0.884 |
| 2024 | Unit 1 | 10 | 0.989 |
| 2024 | Unit 1 | 15 | 0.993 |
| 2024 | Unit 1 | 20 | 0.993 |
| 2024 | Unit 2 | 5 | 0.653 |
| 2024 | Unit 2 | 10 | 0.977 |
| 2024 | Unit 2 | 15 | 0.99 |
| 2024 | Unit 2 | 20 | 0.99 |
| 2024 | Unit 3 | 5 | 0.917 |
| 2024 | Unit 3 | 10 | 0.987 |
| 2024 | Unit 3 | 15 | 0.989 |
| 2024 | Unit 3 | 20 | 0.989 |
| 2024 | Unit 4 | 5 | 0.849 |
| 2024 | Unit 4 | 10 | 0.99 |
| 2024 | Unit 4 | 15 | 0.995 |
| 2024 | Unit 4 | 20 | 0.995 |
| 2024 | Unit 5 | 5 | 0.949 |
| 2024 | Unit 5 | 10 | 0.996 |
| 2024 | Unit 5 | 15 | 0.996 |
| 2024 | Unit 5 | 20 | 0.996 |

3. **Hormone assays**

Plasma samples were extracted with dichloromethane (DCM) after overnight equilibration of the plasma at 4 °C with 1500 dpm of tritiated testosterone (Perkin Elmer, Rodgau, Germany). The organic phase was separated from the aqueous phase by immersing the extraction tubes in a methanol–dry ice bath, after which the DCM layer was decanted into a fresh vial. To enhance extraction efficiency, this procedure was performed twice. The combined DCM extracts were evaporated under a nitrogen stream at 40 °C, and the dried samples were re-suspended in phosphate-buffered saline containing 1 % gelatine (PBSG) and allowed to equilibrate overnight at 4 °C. An aliquot of 80 µl from the re-suspended samples was transferred to scintillation vials, combined with 4 ml scintillation fluid (Packard Ultima Gold), and counted with a Beckman LS 6000 β-counter to an accuracy of 2–3 % in order to determine individual extraction recoveries. The remaining sample volume was stored at −40 °C until RIA analysis. Mean ± SD extraction efficiency for plasma testosterone was 86.7 ± 8.3 %. For the RIA, a standard curve was prepared in duplicate using serial dilutions of stock testosterone standards ranging from 0.39 to 200 pg. Testosterone antiserum (T3-125, Esoterix Endocrinology, Calabasas, CA, USA) was added to the standard curve, controls, and duplicate sample aliquots (100 µl). Cross-reactivities of the antiserum were as follows: testosterone (100 %), 5α-dihydrotestosterone (44 %), d-1-testosterone (41 %), d-1-dihydrotestosterone (18 %), 5α-androstan-3β,17β-diol (3 %), 4-androsten-3β,17β-diol (2.5 %), d-4-androstenedione (2 %), 5β-androstan-3β,17β-diol (1.5 %), estradiol (0.5 %), and less than 0.2 % for 23 additional steroids tested. After 30 min, testosterone label (13 500 dpm) was added, and samples were incubated for 20 h at 4 °C. Separation of bound and free hormone fractions was done at 4 °C by adding 0.5 ml dextran-coated charcoal in PBSG assay buffer. Following a 14-min incubation with charcoal, samples were centrifuged (3600 g, 10 min, 4 °C), and the supernatants were decanted into scintillation vials maintained at 4 °C. After adding 4 ml scintillation liquid (Packard Ultima Gold), vials were counted. Standard curves and testosterone concentrations were calculated using Immunofit 3.0 software (Beckman Inc., Fullerton, CA) with a four-parameter logistic curve fit. The assay detection limit was defined as the first concentration outside the 95 % confidence interval of the zero standard (B_max_) and ranged from 32-40 pg/ml (4 assays in total). The intra-assay coefficient of variation, as determined by standard chicken pool samples extracted with each assay, was 5.0 ± 1.7 %, and inter-assay variation was 2.0 %. Due to substantial cross-reactivity of the antibody with 5α-dihydrotestosterone (44 %), measured testosterone values may include a contribution from 5α-DHT, though ruffs typically do not have a substantial amount of DHT in the blood (Casagrande et al. 2012).

Casagrande, S., Costantini, D., Dell’Omo, G., Tagliavini, J., & Groothuis, T. G. G. (2012). Differential effects of testosterone metabolites oestradiol and dihydrotestosterone on oxidative stress and carotenoid-dependent colour expression in a bird. *Behavioral Ecology and Sociobiology*, 66(9), 1319–1331. https://doi.org/10.1007/s00265-012-1387-3

4. **Detailed statistical analyses**

*Model evaluation*
Model assumptions were assessed via visual inspection of residuals, Q–Q plots, and homoscedasticity across fitted values and random-effect levels. Full models were compared to the respective null models (i.e., control predictors, intercepts and random effects) with a likelihood ratio test. For variables for which we expected a temporal peak (date, age, testosterone – see below) we included quadratic terms. All analyses were conducted in R 3.6.2 (R Core Team 2019).

*Core and total activity area*

We first examined whether the experimental treatment (single vs. mixed morph) had an effect on core and total activity areas (50% and 95% KDEs) across morphs fitting two separate linear mixed-effect models using a Gaussian error distribution, with log-transformed 50% and 95% KDE areas a response variable, and morph, treatment, and their interaction as fixed effects. The interaction term specifically tested whether treatment affected male morphs in different directions. We controlled for potential differences between years by including year as a fixed effect, and included age as a further co-variate. Because observations were repeated within both individuals and specific combinations of unit and year, we included bird ID and a grouping factor for unit-year as random intercepts. To investigate temporal patterns (i.e., changes in core and totally activity areas throughout the breeding season), the ordinal day of the season was centred on the mean and a quadratic term was created to allow for nonlinear seasonal trends. In both models we included the interaction between the linear and quadratic seasonal terms (day), morph, and treatment, yielding a three-way interaction structure. This allowed us to test whether the shape and direction of seasonal changes in area differed among morphs and whether these morph-specific trajectories were affected by the experimental treatment. Bird ID and unit–year were again included as random intercepts. Linear and quadratic seasonal trends were extracted for each morph-treatment combination, and we compared morphs within each treatment and between treatments using estimated marginal trends (‘emtrends’) from the emmeans package (Lenth 2020). For all the above four models, to test whether the interaction term improved explanatory power, we compared the full model to a reduced **null model** lacking the interaction term and containing only additive fixed effects while retaining the random structure; comparisons were performed by likelihood-ratio test using the *anova* function. For the two models with a three-way interaction term, we also compared each three-way model to a null model containing only two-way interactions. Assumptions of linear mixed-effects modelling were evaluated using visual inspection of fitted residuals, Q–Q plots, and assessments of homoscedasticity across fitted values and across levels of the random effects. All models were fitted using the *lmer* function in the *lme4* package (Bates et al. 2015).

*Pairwise centroid distances*

We modelled the pairwise Euclidean distance between centroids calculated for each dyad of birds within a given session and unit, and for both 50% and 95% KDE contours. Inter-centroid distances were positive and right-skewed; therefore, we modelled them using a lognormal error distribution. Our primary predictor was **dyad type**, which classified each pair according to the morph combination of the two individuals and whether they occurred in a single- or mixed-morph treatment [e.g. Sat-Sat (Single), Sat-Ind (Mixed) etc.]. To account for non-independence arising from repeated measurements of individuals and dyads, we fitted a multilevel model with the following random effects: (i) **individual identity**, modelled as a multi-membership random intercept to account for the fact that each observation involved two individuals, which allowed both individuals in a dyad to contribute equally to variance in pairwise distance; (ii) **dyad identity**, included as a random intercept to account for repeated measurements of the same pair across days; unit-year as described above. Models were fitted using the ***brms*** package (Bürkner 2021). Model convergence and adequacy were assessed using standard diagnostic criteria, including R̂, effective sample sizes, and posterior predictive checks. Pairwise comparisons among dyad types were conducted using estimated marginal means (package *emmeans*) (Lenth 2020). Random effects indicated substantial variance attributable to dyad identity (SD = 0.27) and individual identity (multi-membership: KDE 50% SD = 0.25; KDE 95% SD = 0.31), suggesting consistent pair- and individual-level differences in spatial distancing.

For the analysis of within-individual repeatability in space use, we modelled the Euclidean distance between centroids of the same male between consecutive sessions. Fixed effects included treatment group (single vs. mixed), morph type, and their interaction, as well as the covariates year and age. We included random intercepts for individual identity (Bird ID) and unit within year (unit-year). Model assumptions and full-null model comparisons were implemented as described above.

*Overlap proportions*

For both KDE contours (50% and 95%), we fitted overlap proportions (bound between 0-1) as a function of morph, treatment, and their interaction. We included age as a co-variate, whereas bird ID, partner ID, and unit-year were fitted as random intercepts. Both models were fitted with a beta distribution using the *glmmTMB* package (Brooks et al. 2017). Because of the large proportion of zeros (~38%) in the dataset for 50% KDEs, this model was additionally fitted with a ‘zero-inflation’ component. Full-null model comparisons, model stability, diagnostics, and confidence intervals were extracted using the same methods described above. Overdispersion was evaluated using the *DHaRMa* package (Hartig 2024). We investigated temporal patterns in territory overlap by modelling the quadratic term for date as described above, fitting two models for the 50% and 95% KDE contours with a beta distribution using the *glmmTMB* package (Brooks et al. 2017). As a validity check, pairwise spatial overlap between individuals was additionally quantified using an alternative overlap index commonly used to compute overlap between KDEs: Utilization Distribution Overlap (UDOI) (Fieberg and Kochanny 2010). **Unlike proportional overlap,** this index incorporates the full utilization distributions, reflects the degree of space-use sharing and is sensitive to how intensively individuals use the same areas. This metric was derived using raster-based integration of overlapping utilization distribution values. For each session and unit UDOI values were computed across all pairs of male ruffs. We analysed variation in utilization overlap among dyad types using a Bayesian multilevel model implemented in *brms* (Bürkner 2021). UDOI was modelled using a Beta error distribution with logit link. The main predictor of interest was dyad type (categorical factor with levels: Fae–Fae, Fae–Ind, Fae–Sat, Ind–Ind, Ind–Sat, Sat–Sat). Year was included as fixed effect. To account for non-independence in the data, we included multiple random effects: a random intercept for unit-year to account for repeated observations within units across years; a random intercept for day-within-year; a multi-membership random effect for individuals to account for dyadic dependence. For both models, we used weakly informative priors. Models were fit using four Markov chain Monte Carlo (MCMC) chains with 4000 iterations each (1000 warm-up), yielding 12,000 posterior samples. The results obtained with these two overlap metrics (proportional overlap and UDOI indexes) did not differ substantially (see below), therefore only the proportional results models are shown in the main text.

*Testosterone levels*

We modelled log-transformed testosterone concentrations as a function of morph and age (both the linear and quadratic term, allowing for non-linear age effects) to test whether the relationship between age and testosterone levels differed among morphs. The full interaction model (i.e. morph:age) was compared with a reduced model without the interaction term using *anova*. As the interaction was not significant, the reduced model was retained. We then tested whether inclusion of a quadratic age term improved model fit by comparing models with linear versus quadratic age terms. Since testosterone was only analysed in 2024, we only included Bird ID as random intercept. Finally, to investigate whether testosterone levels related to space use measures (for both 50% and 95% KDEs), we ran separate models with either areas, inter-centroid distances and proportional overlap values as response variables and an interaction term between testosterone concentration and morph as predictor.

*Spatial distribution of copulations*

We modelled the probability that a copulation occurred within the 50% and 95% KDE polygons using generalized linear mixed models with binomial error distribution and logit link function. Fixed effects included the interaction between male morph and treatment, and the area of the KDE polygon to account for differences in core and total activity areas among males (see Results). Male identity was included as a random intercept to account for repeated copulations per individual. To test for differences in the spatial dispersion of copulations among morphs, we modelled convex hull areas using linear mixed effects models with morph as a fixed effect and bird identity as a random intercept.

Bates, D., M. Mächler, B. Bolker, and S. Walker. (2015). Fitting linear mixed-effects models using lme4. Journal of Statistical Software 67(1), 1–48. doi:10.18637/jss.v067.i01

Brooks, M. E., K. Kristensen, K. J. V. Benthem, A. Magnusson, C. W. Berg, A. Nielsen, et al. (2017). glmmTMB balances speed and flexibility among packages for zero-inflated gen- eralized linear mixed modeling. The R Journal 9(2), 378–400. doi:10.32614/RJ-2017-066

Bürkner, P.-C. (2021). Bayesian Item Response Modeling in R with brms and Stan. Journal of Statistical Software, 100, 1–54. <https://doi.org/10.18637/jss.v100.i05>

Fieberg, J., & Kochanny, C. O. (2005). Quantifying Home-Range Overlap: The Importance of the Utilization Distribution. The Journal of Wildlife Management, 69(4), 1346–1359. https://doi.org/10.2193/0022-541X(2005)69[1346:QHOTIO]2.0.CO;2

Hartig, F. (2024). DHARMa: Residual Diagnostics for Hierarchical (Multi-Level / Mixed) Regression Models_. R package version 0.4.

Lenth, R. 2020. ‘Emmeans: Estimated Marginal Means, Aka Least- Squares Means.’ https://CRAN.R-project.org/pack age=emmeans

R Core Team (2019). 'R: A Language and Environment for Statistical Computing. R Foundation for Statistical Computing, Vienna, Austria. http://www.R-project.org

**Results of the models for Utilization Distribution Overlap (UDOI)**

Dyad type had a clear effect on utilization overlap (UDOI). Faeder–Faeder and Satellite–Satellite dyads exhibited the highest overlap (Figure S2), which did not differ credibly from one another (odds ratio = 0.68, 95% HPD: 0.24–1.29). Both of these dyad types exhibited higher overlap than several mixed dyads. Similarly, Independent–Independent dyads showed lower overlap than Independent–Satellite (odds ratio = 0.64, 95% HPD: 0.52–0.78) and Satellite–Satellite dyads (odds ratio = 0.28, 95% HPD: 0.12–0.55). Among mixed dyads, overlap tended to be lower overall and several contrasts involving intermediate dyad types (e.g., Fae–Sat vs. Ind–Sat, Fae–Fae vs. Fae–Sat) were uncertain, with HPD intervals overlapping 1.

**Figure S2.** Predicted utilization overlap across dyad types. Points represent posterior median estimates from a Bayesian multilevel model, and vertical bars indicate 66% (thick lines) and 95% (thin lines) credible intervals. Predictions are shown on the response scale and averaged across levels of year and random effects. Dyad types differ in overlap, with highest values observed for same-type dyads (Fae–Fae and Sat–Sat) and generally lower values for mixed dyads.

**Figure S3.** Pairwise comparisons of utilization overlap among dyad types based on posterior estimates from a Bayesian multilevel model. Points represent median differences between dyad types, and horizontal bars indicate 95% credible intervals. Intervals that do not overlap zero indicate credible differences between dyad types. Positive values indicate higher overlap in the first dyad relative to the second.

# Table S3. Results of linear mixed model for KDE 50% area

## (A) Fixed effects

| Variable | Estimate | 95% CI lower | 95% CI upper | SE | df | p value |
| --- | --- | --- | --- | --- | --- | --- |
| Intercept | 10.156 | 9.630 | 10.706 | 0.269 | 16.14 | - |
| Treatment (mixed) | -0.177 | -0.717 | 0.319 | 0.264 | 10.84 | 0.518 |
| Morph (Ind) | -1.141 | -1.779 | -0.491 | 0.328 | 13.59 | 0.004 |
| Morph (Sat) | 0.468 | -0.226 | 1.118 | 0.335 | 14.47 | 0.184 |
| Year (2024) | 0.369 | 0.022 | 0.714 | 0.179 | 8.00 | 0.073 |
| Age | -0.030 | -0.076 | 0.019 | 0.025 | 32.98 | 0.225 |
| Treatment × Morph (Ind) | 0.992 | 0.381 | 1.612 | 0.312 | 11.88 | 0.008 |
| Treatment × Morph (Sat) | -0.057 | -0.724 | 0.588 | 0.315 | 12.38 | 0.859 |

## (B) Morph contrasts within Treatment

| Treatment | Contrast | Estimate | SE | df | t ratio | p value |
| --- | --- | --- | --- | --- | --- | --- |
| single | Fae − Ind | 1.141 | 0.450 | 29.9 | 2.532 | 0.043 |
| single | Fae − Sat | -0.468 | 0.457 | 32.3 | -1.025 | 0.567 |
| single | Ind − Sat | -1.609 | 0.449 | 30.9 | -3.582 | 0.003 |
| mixed | Fae − Ind | 0.148 | 0.221 | 58.1 | 0.672 | 0.781 |
| mixed | Fae − Sat | -0.411 | 0.250 | 63.1 | -1.641 | 0.236 |
| mixed | Ind − Sat | -0.559 | 0.215 | 55.1 | -2.596 | 0.032 |

## (C) Treatment contrasts within Morph

| Treatment | Contrast | Estimate | SE | df | t ratio | p value |
| --- | --- | --- | --- | --- | --- | --- |
| Fae | single − mixed | 0.177 | 0.372 | 23.4 | 0.474 | 0.640 |
| Ind | single − mixed | -0.815 | 0.362 | 21.4 | -2.251 | 0.035 |
| Sat | single − mixed | 0.234 | 0.365 | 22.4 | 0.641 | 0.528 |

# Table S4. Results of linear mixed model for KDE 95% area

## (A) Fixed effects

| Term | Estimate | 95% CI lower | 95% CI upper | SE | df | p value |
| --- | --- | --- | --- | --- | --- | --- |
| Intercept | 11.878 | 11.451 | 12.373 | 0.244 | 15.53 | - |
| Treatment (mixed) | -0.162 | -0.653 | 0.291 | 0.242 | 10.57 | 0.516 |
| Morph (Ind) | -1.088 | -1.684 | -0.527 | 0.300 | 13.33 | 0.003 |
| Morph (Sat) | 0.269 | -0.310 | 0.817 | 0.306 | 14.16 | 0.395 |
| Year (2024) | 0.336 | 0.027 | 0.665 | 0.167 | 8.44 | 0.077 |
| Age | -0.031 | -0.077 | 0.007 | 0.021 | 33.00 | 0.148 |
| Treatment × Morph (Ind) | 0.956 | 0.391 | 1.571 | 0.284 | 11.34 | 0.006 |
| Treatment × Morph (Sat) | 0.063 | -0.498 | 0.642 | 0.286 | 11.67 | 0.829 |

# (B) Morph contrasts within Treatment

| Treatment | Contrast | Estimate | SE | df | t ratio | p value |
| --- | --- | --- | --- | --- | --- | --- |
| single | Fae − Ind | 1.088 | 0.412 | 28.9 | 2.637 | 0.035 |
| single | Fae − Sat | -0.269 | 0.418 | 31.2 | -0.644 | 0.797 |
| single | Ind − Sat | -1.356 | 0.411 | 29.8 | -3.297 | 0.007 |
| mixed | Fae − Ind | 0.132 | 0.186 | 56.4 | 0.708 | 0.760 |
| mixed | Fae − Sat | -0.332 | 0.210 | 60.6 | -1.580 | 0.262 |
| mixed | Ind − Sat | -0.464 | 0.182 | 53.5 | -2.552 | 0.036 |

# (C) Treatment contrasts within Morph

| Treatment | Contrast | Estimate | SE | df | t ratio | p value |
| --- | --- | --- | --- | --- | --- | --- |
| Fae | single − mixed | 0.162 | 0.341 | 22.4 | 0.476 | 0.639 |
| Ind | single − mixed | -0.793 | 0.334 | 21.1 | -2.376 | 0.027 |
| Sat | single − mixed | 0.099 | 0.336 | 21.7 | 0.295 | 0.771 |

# Table S5. Models for seasonal trends of KDE 50% area (log-transformed)

## (A) Three-way interaction model

| Term | Estimate | CI lower | CI upper | SE | df | p value |
| --- | --- | --- | --- | --- | --- | --- |
| Intercept | 10.193 | 9.797 | 10.551 | 0.197 | 11.6 | - |
| Date (centred) | 0.010 | -0.003 | 0.021 | 0.00619 | 1237 | 0.120 |
| Date^2^ (centred) | -0.001 | -0.002 | 0.001 | 0.000715 | 1235 | 0.388 |
| MorphInd | -1.243 | -1.778 | -0.692 | 0.277 | 11.3 | <0.001 |
| MorphSat | 0.564 | 0.046 | 1.139 | 0.278 | 11.3 | 0.066 |
| Treatmentmixed | 0.167 | -0.467 | 0.836 | 0.330 | 34.7 | 0.616 |
| Date × MorphInd | -0.017 | -0.033 | -0.001 | 0.00824 | 1236 | 0.038 |
| Date × MorphSat | 0.010 | -0.008 | 0.026 | 0.00850 | 1238 | 0.245 |
| Date^2^× MorphInd | 0.004 | 0.002 | 0.006 | 0.00100 | 1235 | <0.001 |
| Date^2^× MorphSat | -0.001 | -0.003 | 0.001 | 0.00103 | 1237 | 0.597 |
| Date × EUmixed | -0.004 | -0.026 | 0.016 | 0.0112 | 1237 | 0.735 |
| Date^2^ × EUmixed | 0.002 | -0.001 | 0.004 | 0.00139 | 1235 | 0.258 |
| Date × MorphInd × EUmixed | 0.018 | -0.008 | 0.045 | 0.0135 | 1236 | 0.193 |
| Date^2^× MorphInd × EUmixed | -0.003 | -0.006 | 0.000 | 0.00168 | 1235 | 0.088 |

# (B) Model for Independents (reduced model)

| Term | Estimate | CI lower | CI upper | SE | df | p value |
| --- | --- | --- | --- | --- | --- | --- |
| Intercept | 8.999 | 8.541 | 9.361 | 0.207 | 24.0 | - |
| Date (centred) | -0.001 | -0.021 | 0.006 | 0.00469 | 580 | 0.901 |
| Date² (centred) | 0.002 | 0.002 | 0.005 | 0.000597 | 577 | <0.001 |
| Treatment:mixed | 0.566 | 0.118 | 1.221 | 0.275 | 22.1 | 0.052 |

# (C) Satellites (full model)

| Term | Estimate | CI lower | CI upper | SE | df | p value |
| --- | --- | --- | --- | --- | --- | --- |
| Intercept | 10.760 | 10.606 | 10.907 | 0.0775 | 19.4 | - |
| Date (centred) | 0.020 | 0.013 | 0.026 | 0.00341 | 360 | <0.001 |
| Date² (centred) | -0.001 | -0.002 | 0.000 | 0.000433 | 362 | 0.009 |
| Treatment:mixed | -0.327 | -0.605 | -0.020 | 0.145 | 19.3 | 0.036 |
| Date × Tratment:mixed | -0.019 | -0.031 | -0.007 | 0.00608 | 359 | 0.002 |
| Date^2^ × Treatment:mixed | 0.002 | 0.000 | 0.003 | 0.000779 | 360 | 0.040 |

# (D) Faeders (full model)

| Term | Estimate | CI lower | CI upper | SE | df | p value |
| --- | --- | --- | --- | --- | --- | --- |
| Intercept | 10.19 | — | — | 0.147 | 17.1 | - |
| Date (centred) | 0.010 | — | — | 0.00503 | 311 | 0.056 |
| Date² (centred) | -0.001 | — | — | 0.000581 | 311 | 0.286 |
| Treatmentmixed | 0.169 | — | — | 0.273 | 16.6 | 0.544 |
| Date × Tratment:mixed | -0.004 | — | — | 0.00911 | 311 | 0.659 |
| Date^2^ × Treatment:mixed | 0.002 | — | — | 0.00113 | 310 | 0.167 |

# Table S6. Three-way interaction model for KDE 95% area

## (A) Fixed effects

| Term | Estimate | CI lower | CI upper | SE | df | p value |
| --- | --- | --- | --- | --- | --- | --- |
| Intercept | 11.885 | 11.547 | 12.221 | 0.175 | 11.2 | - |
| Date (centred) | 0.005 | -0.005 | 0.014 | 0.00483 | 1236 | 0.337 |
| Date^2^ (centred) | -0.001 | -0.002 | 0.001 | 0.000558 | 1235 | 0.358 |
| MorphInd | -1.158 | -1.667 | -0.709 | 0.247 | 11.0 | <0.001 |
| MorphSat | 0.371 | -0.090 | 0.892 | 0.247 | 11.0 | 0.161 |
| Treatmentmixed | 0.093 | -0.505 | 0.659 | 0.286 | 31.0 | 0.746 |
| Date × MorphInd | -0.016 | -0.028 | -0.003 | 0.00642 | 1235 | 0.014 |
| Date × MorphSat | 0.008 | -0.004 | 0.021 | 0.00663 | 1237 | 0.214 |
| Date^2^× MorphInd | 0.004 | 0.002 | 0.005 | 0.000777 | 1235 | <0.001 |
| Date^2^× MorphSat | -0.001 | -0.002 | 0.001 | 0.000803 | 1237 | 0.477 |
| Date × EUmixed | 0.001 | -0.016 | 0.018 | 0.00873 | 1237 | 0.944 |
| Date^2^× EUmixed | 0.001 | -0.001 | 0.003 | 0.00108 | 1235 | 0.208 |
| Date × MorphInd × EUmixed | 0.015 | -0.005 | 0.036 | 0.0105 | 1236 | 0.157 |
| Date^2^× MorphInd × EUmixed | -0.002 | -0.005 | 0.000 | 0.00131 | 1235 | 0.061 |

# (B) Independents (reduced models)

| Term | Estimate | CI lower | CI upper | SE | df | p |
| --- | --- | --- | --- | --- | --- | --- |
| Intercept | 10.77 | 10.39 | 11.143 | 0.191 | 23.6 | <0.001 |
| Date | -0.003 | -0.011 | 0.005 | 0.00391 | 579 | 0.409 |
| Date2 | 0.003 | 0.002 | 0.003 | 0.000498 | 577 | <0.001 |
| Treatment:mixed | 0.511 | -0.003 | 1.001 | 0.254 | 22.1 | 0.057 |

# (C) Satellites (full model)

| Term | Estimate | CI lower | CI upper | SE | df | p |
| --- | --- | --- | --- | --- | --- | --- |
| Intercept | 12.257 | 12.146 | 12.359 | 0.0565 | 16.9 | <0.001 |
| Date | 0.013 | 0.009 | 0.016 | 0.00197 | 359 | <0.001 |
| Date2 | -0.001 | -0.002 | -0.001 | 0.00025 | 360 | <0.001 |
| Treatmentmixed | -0.213 | -0.413 | 0.001 | 0.106 | 16.8 | 0.060 |
| Date×Treatment | -0.018 | -0.025 | -0.011 | 0.00351 | 358 | <0.001 |
| Date2×Treatment | 0.002 | 0.001 | 0.002 | 0.00045 | 359 | <0.001 |

# (D) Faeders (full model)

| Term | Estimate | CI lower | CI upper | SE | df | p |
| --- | --- | --- | --- | --- | --- | --- |
| Intercept | 11.89 | - | - | 0.0915 | 17.2 | <0.001 |
| Date | 0.0046 | - | - | 0.00316 | 311 | 0.147 |
| Date2 | -0.00052 | - | - | 0.000365 | 310 | 0.157 |
| Treatmentmixed | 0.096 | - | - | 0.17 | 16.8 | 0.580 |
| Date×Treatment | 0.00048 | - | - | 0.00571 | 311 | 0.933 |
| Date2×Treatment | 0.00135 | - | - | 0.000706 | 310 | 0.056 |

Figure S4 (A) Centroid distances of lekking ruffs for pairwise comparisons of morphs show morph specific differences related to stability of territoriality. Posterior predictions of centroid distance (top: 50% KDE; bottom: 95% KDE) as a function of dyad type. Points represent posterior medians of the expected distance on the response scale, obtained from a Bayesian lognormal mixed-effects model. Horizontal lines indicate credible intervals. The model includes dyad type as a fixed effect and random intercepts for individual identity (Bird1 and Bird2), dyad identity, and year. (B) Inter-centroid displacement among morphs across treatment types. Model-derived estimated marginal means ± 95% confidence intervals are shown as points and error bars, respectively.

# Table S7. Bayesian model results for inter-centroid distance of 50% KDEs

## (A) Fixed effects effects (posterior estimates, 95% HPD intervals)

| Term | Estimate | Est.Error | lower 95% HPD | upper 95% HPD |
| --- | --- | --- | --- | --- |
| Intercept | 4.54 | 0.13 | 4.31 | 4.80 |
| Fae_Ind_mixed | 0.53 | 0.16 | 0.20 | 0.82 |
| Fae_Sat_mixed | 0.53 | 0.20 | 0.14 | 0.91 |
| Ind_Ind_mixed | 0.63 | 0.17 | 0.27 | 0.96 |
| Ind_Ind_single | 0.71 | 0.18 | 0.34 | 1.05 |
| Ind_Sat_mixed | 0.44 | 0.17 | 0.09 | 0.77 |
| Sat_Sat_single | 0.19 | 0.17 | -0.16 | 0.52 |

# (B) Random effects (standard deviations)

| Group | SD | lower 95% HPD | upper 95% HPD |
| --- | --- | --- | --- |
| dyad_id | 0.27 | 0.22 | 0.34 |
| EU_year | 0.07 | 0.00 | 0.23 |
| Bird (mm) | 0.25 | 0.03 | 0.42 |

# (C) Estimated marginal means (response scale)

| Dyad type | Estimate | lower 95% HPD | upper 95% HPD |
| --- | --- | --- | --- |
| Fae_Fae_single | 4.54 | 4.30 | 4.80 |
| Fae_Ind_mixed | 5.07 | 4.84 | 5.32 |
| Fae_Sat_mixed | 5.07 | 4.74 | 5.41 |
| Ind_Ind_mixed | 5.17 | 4.92 | 5.41 |
| Ind_Ind_single | 5.26 | 5.01 | 5.51 |
| Ind_Sat_mixed | 4.98 | 4.76 | 5.22 |
| Sat_Sat_single | 4.74 | 4.49 | 4.97 |

# Table S8. Bayesian model results for inter-centroid distance of 95% KDEs

## (A) Fixed effects (posterior estimates, 95% HPD intervals)

| Term | Estimate | Est.Error | lower 95% HPD | upper 95% HPD |
| --- | --- | --- | --- | --- |
| Intercept | 4.03 | 0.15 | 3.76 | 4.34 |
| Fae_Ind_mixed | 0.61 | 0.17 | 0.26 | 0.93 |
| Fae_Sat_mixed | 0.38 | 0.22 | -0.06 | 0.79 |
| Ind_Ind_mixed | 0.81 | 0.20 | 0.40 | 1.18 |
| Ind_Ind_single | 0.94 | 0.21 | 0.49 | 1.31 |
| Ind_Sat_mixed | 0.47 | 0.19 | 0.09 | 0.82 |
| Sat_Sat_single | -0.04 | 0.20 | -0.46 | 0.34 |

# (B) Random effects (standard deviations)

| Group | SD | lower 95% HPD | upper 95% HPD |
| --- | --- | --- | --- |
| dyad_id | 0.27 | 0.21 | 0.34 |
| EU_year | 0.09 | 0.00 | 0.28 |
| Bird (mm) | 0.31 | 0.10 | 0.49 |

# (C) Estimated marginal means (response scale)

| Dyad type | Estimate | lower 95% HPD | upper 95% HPD |
| --- | --- | --- | --- |
| Fae_Fae_single | 4.03 | 3.76 | 4.33 |
| Fae_Ind_mixed | 4.64 | 4.38 | 4.91 |
| Fae_Sat_mixed | 4.41 | 4.04 | 4.79 |
| Ind_Ind_mixed | 4.84 | 4.57 | 5.12 |
| Ind_Ind_single | 4.98 | 4.67 | 5.25 |
| Ind_Sat_mixed | 4.50 | 4.25 | 4.77 |
| Sat_Sat_single | 3.99 | 3.70 | 4.27 |

# Table S9. Linear mixed model results for centroid displacement (within-individual)

## (A) Fixed effects

| Term | Estimate | CI lower | CI upper | SE | df | p value |
| --- | --- | --- | --- | --- | --- | --- |
| Intercept | 4.304 | 4.062 | 4.559 | 0.121 | 17.4 | - |
| Treatment (mixed) | 0.040 | -0.226 | 0.314 | 0.141 | 29.9 | 0.780 |
| Morph (Ind) | -0.384 | -0.649 | -0.099 | 0.138 | 12.9 | 0.015 |
| Morph (Sat) | 0.195 | -0.075 | 0.495 | 0.140 | 12.9 | 0.187 |
| Year (2024) | 0.137 | 0.002 | 0.261 | 0.065 | 5.4 | 0.085 |
| Age | -0.002 | -0.035 | 0.031 | 0.016 | 27.1 | 0.879 |
| Treatment × Morph (Ind) | 0.389 | 0.059 | 0.703 | 0.176 | 39.9 | 0.032 |
| Treatment × Morph (Sat) | -0.029 | -0.384 | 0.328 | 0.190 | 54.9 | 0.880 |

# (B) Morph contrasts within Treatment

| Treatment | Contrast | Estimate | SE | df | t ratio | p value |
| --- | --- | --- | --- | --- | --- | --- |
| single | Fae − Ind | 0.384 | 0.190 | 28.9 | 2.019 | 0.126 |
| single | Fae − Sat | -0.195 | 0.192 | 31.3 | -1.013 | 0.574 |
| single | Ind − Sat | -0.579 | 0.186 | 29.7 | -3.108 | 0.011 |
| mixed | Fae − Ind | -0.005 | 0.172 | 84.4 | -0.030 | 0.999 |
| mixed | Fae − Sat | -0.166 | 0.201 | 101.7 | -0.824 | 0.689 |
| mixed | Ind − Sat | -0.161 | 0.167 | 86.5 | -0.962 | 0.603 |

# (C) Treatment contrasts within Morph

| Treatment | Contrast | Estimate | SE | df | t ratio | p value |
| --- | --- | --- | --- | --- | --- | --- |
| Fae | single − mixed | -0.040 | 0.180 | 52.4 | -0.220 | 0.827 |
| Ind | single − mixed | -0.429 | 0.151 | 26.5 | -2.836 | 0.009 |
| Sat | single − mixed | -0.011 | 0.167 | 47.7 | -0.065 | 0.949 |

# Table S10. Zero-inflated beta mixed model results for KDE 50% overlap

## (A) Conditional model

| Term | Estimate | SE | z value | p value |
| --- | --- | --- | --- | --- |
| Intercept | -0.729 | 0.149 | -4.899 | - |
| Morph (Ind) | -0.205 | 0.161 | -1.271 | 0.204 |
| Morph (Sat) | -0.089 | 0.179 | -0.499 | 0.618 |
| Treatment (single) | 0.255 | 0.132 | 1.936 | 0.053 |
| Age | -0.004 | 0.018 | -0.243 | 0.808 |
| Year (2024) | -0.046 | 0.067 | -0.697 | 0.486 |
| MorphInd × EU | -0.404 | 0.211 | -1.918 | 0.055 |
| MorphSat × EU | -0.077 | 0.176 | -0.437 | 0.662 |

# (B) Zero-inflation model (probability of zero overlap)

| Term | Estimate | SE | z value | p value |
| --- | --- | --- | --- | --- |
| Intercept | -0.716 | 0.107 | -6.666 | <0.001 |
| Morph (Ind) | 0.665 | 0.122 | 5.461 | <0.001 |
| Morph (Sat) | -0.053 | 0.150 | -0.353 | 0.724 |
| Treatment (single) | -1.073 | 0.145 | -7.385 | <0.001 |
| MorphInd × EU | 2.455 | 0.173 | 14.223 | <0.001 |
| MorphSat × EU | -0.104 | 0.203 | -0.512 | 0.609 |

# (C) Conditional contrasts (Morph within Treatment)

| Treatment | Contrast | Estimate | SE | z ratio | p value |
| --- | --- | --- | --- | --- | --- |
| mixed | Fae − Ind | 0.205 | 0.161 | 1.271 | 0.412 |
| mixed | Fae − Sat | 0.089 | 0.179 | 0.499 | 0.872 |
| mixed | Ind − Sat | -0.116 | 0.157 | -0.741 | 0.739 |
| single | Fae − Ind | 0.609 | 0.199 | 3.062 | 0.006 |
| single | Fae − Sat | 0.166 | 0.174 | 0.956 | 0.605 |
| single | Ind − Sat | -0.443 | 0.194 | -2.281 | 0.059 |

# (D) Zero-inflation contrasts (Morph within Treatment)

| Treatment | Contrast | Estimate | SE | z ratio | p value |
| --- | --- | --- | --- | --- | --- |
| mixed | Fae − Ind | -0.665 | 0.122 | -5.461 | <0.001 |
| mixed | Fae − Sat | 0.053 | 0.150 | 0.353 | 0.934 |
| mixed | Ind − Sat | 0.718 | 0.120 | 6.006 | <0.001 |
| single | Fae − Ind | -3.120 | 0.122 | -25.505 | <0.001 |
| single | Fae − Sat | 0.157 | 0.137 | 1.149 | 0.484 |
| single | Ind − Sat | 3.277 | 0.120 | 27.242 | <0.001 |

# Table S11. Beta mixed model results for KDE 95% overlap

## (A) Fixed effects

| Term | Estimate | SE | z value | p value |
| --- | --- | --- | --- | --- |
| Intercept | 0.139 | 0.278 | 0.500 | 0.617 |
| Morph (Ind) | -0.396 | 0.157 | -2.516 | 0.012 |
| Morph (Sat) | -0.248 | 0.178 | -1.390 | 0.164 |
| Treatment (single) | 0.637 | 0.342 | 1.864 | 0.062 |
| Age | -0.012 | 0.019 | -0.643 | 0.520 |
| Year (2024) | 0.480 | 0.235 | 2.041 | 0.041 |
| MorphInd × Treatment | -1.410 | 0.402 | -3.511 | <0.001 |
| MorphSat × Treatment | -0.395 | 0.404 | -0.977 | 0.329 |

# (B) Morph contrasts within Treatment

| Treatment | Contrast | Estimate | SE | z ratio | p value |
| --- | --- | --- | --- | --- | --- |
| mixed | Fae − Ind | 0.396 | 0.157 | 2.516 | 0.032 |
| mixed | Fae − Sat | 0.248 | 0.178 | 1.390 | 0.346 |
| mixed | Ind − Sat | -0.148 | 0.153 | -0.962 | 0.601 |
| single | Fae − Ind | 1.805 | 0.405 | 4.460 | <0.001 |
| single | Fae − Sat | 0.643 | 0.408 | 1.575 | 0.257 |
| single | Ind − Sat | -1.163 | 0.400 | -2.907 | 0.010 |

# (C) Treatment contrasts within Morph

| Morph | Contrast | Estimate | SE | z ratio | p value |
| --- | --- | --- | --- | --- | --- |
| Fae | mixed − single | -0.637 | 0.342 | -1.864 | 0.062 |
| Ind | mixed − single | 0.772 | 0.328 | 2.353 | 0.019 |
| Sat | mixed − single | -0.243 | 0.332 | -0.732 | 0.464 |

# Table S12. Model summaries for three models using KDE 50% contour levels and relationship with testosterone concentration

(A) Area

| Predictor | Estimate | SE | Test | p |
| --- | --- | --- | --- | --- |
| Intercept | 10.048 | 0.176 | t=57.06 | <0.001 |
| Log_concent | -0.002 | 0.085 | t=-0.03 | 0.978 |
| MorphInd | -0.212 | 0.222 | t=-0.96 | 0.342 |
| MorphSat | 0.623 | 0.225 | t=2.77 | 0.007 |

# (B) Overlap

| Predictor | Estimate | SE | Test | p |
| --- | --- | --- | --- | --- |
| Intercept | -1.352 | 0.145 | z=-9.35 | <0.001 |
| Log_concent | -0.029 | 0.057 | z=-0.52 | 0.603 |
| MorphInd | -0.520 | 0.171 | z=-3.05 | 0.002 |
| MorphSat | 0.359 | 0.182 | z=1.98 | 0.048 |

# (C) Inter-centroid distance

| Predictor | Estimate | SE | Test | p |
| --- | --- | --- | --- | --- |
| Intercept | 141.662 | 16.443 | t=8.62 | <0.001 |
| Log_concent | -6.959 | 4.441 | t=-1.57 | 0.118 |
| MorphInd | 85.159 | 21.034 | t=4.05 | <0.001 |
| MorphSat | 9.943 | 22.571 | t=0.44 | 0.663 |

# Table S13. Model summaries for three models using KDE 95% contour levels and relationship with testosterone concentration

(A) Area

| Predictor | Estimate | SE | Test | p |
| --- | --- | --- | --- | --- |
| Intercept | 11.863 | 0.120 | t=98.72 | <0.001 |
| Log_concent | -0.006 | 0.059 | t=-0.10 | 0.920 |
| MorphInd | -0.279 | 0.151 | t=-1.85 | 0.068 |
| MorphSat | 0.296 | 0.153 | t=1.93 | 0.056 |

# (B) Overlap

| Predictor | Estimate | SE | Test | p |
| --- | --- | --- | --- | --- |
| Intercept | 0.665 | 0.199 | z=3.35 | <0.001 |
| Log_concent | -0.001 | 0.060 | z=-0.02 | 0.982 |
| MorphInd | -0.566 | 0.254 | z=-2.23 | 0.026 |
| MorphSat | -0.068 | 0.269 | z=-0.25 | 0.799 |

# (C) Inter-centroid distance

| Predictor | Estimate | SE | Test | p |
| --- | --- | --- | --- | --- |
| Intercept | 80.022 | 12.141 | t=6.59 | <0.001 |
| Log_concent | -2.040 | 3.028 | t=-0.67 | 0.501 |
| MorphInd | 62.013 | 15.533 | t=3.99 | <0.001 |
| MorphSat | -1.183 | 16.741 | t=-0.07 | 0.944 |

| Sat–Sat (single) | 0.19 | 0.19 |
| --- | --- | --- |
| sd(dyad_id) | 0.27 | 0.28 |
| sd(mmBird1Bird2) | 0.25 | 0.25 |
| sd(EU_year) | 0.07 | 0.07 |
| Residual (sigma) | 0.60 | 0.60 |

Figure S5 Number of copulations observed within each experimental unit during each session in the two lekking seasons (2023 and 2024)

Figure S6 Plasma testosterone concentrations (pg/mL) of male ruffs during the lekking season. Solid lines connect testosterone levels of the same individual.

**Results of model comparisons using truncated and untruncated datasets**

Reanalysis using the truncated dataset (i.e., excluding observations after group size reduction in 2024) yielded comparable results. In the model for 50% KDE area, the interaction between morph and experimental unit type remained significant (χ² = 8.40, p = 0.015), and effect sizes were comparable to those obtained from the full dataset (Table S14). The three-way interaction between time, morph, and experimental unit type was significant in the full dataset (χ² = 11.12, p = 0.025), but not in the truncated dataset (χ² = 6.33, p = 0.176) (Table S15). However, all lower-order effects and temporal patterns remained qualitatively unchanged. In the model for KDE overlap values, the zero-inflation component remained strongly structured by morph and experimental unit type, with a significant interaction between the two (p < 0.001), while effects in the conditional model remained weak (Table S16). In the model for centroid distance, wstimates, credible intervals, and pairwise contrasts were nearly identical between full and truncated datasets (Table S17), indicating that variation in group size did not influence centroid distances.

**Table S14. Comparison of model estimates between full and truncated datasets (50% KDE area)**

| Parameter | Full dataset | Truncated dataset |
| --- | --- | --- |
| Intercept | 10.156 | 10.159 |
| Treatment (mixed) | -0.177 | -0.107 |
| Morph (Ind) | -1.141 | -1.109 |
| Morph (Sat) | 0.468 | 0.446 |
| Year (2024) | 0.369 | 0.348 |
| Age | -0.030 | -0.031 |
| Treatment × Morph (Ind) | 0.992 | 0.862 |
| Treatment × Morph (Sat) | -0.057 | -0.124 |
| Interaction test (χ², p) | 9.32, 0.009 | 8.40, 0.015 |

**Table S15. Comparison of 3-way model results (full vs truncated datasets)**

| Component | Full dataset | Truncated dataset |
| --- | --- | --- |
| Full vs null (χ², p) | 39.14, <0.001 | 36.62, <0.001 |
| 3-way interaction test (χ², p) | 11.12, 0.025 | 6.33, 0.176 |
| Morph (Ind) | -1.243 | -1.232 |
| Morph (Sat) | 0.564 | 0.591 |
| DayNum × Morph (Ind) | -0.017 | -0.021 |
| DayNum² × Morph (Ind) | 0.00377 | 0.00377 |
| Key pattern: Ind quadratic effect | Significant | Significant |
| Key pattern: Sat linear (single EU) | Positive | Positive |

**Table S16. Comparison of zero-inflated beta model (full vs truncated datasets)**

| Parameter | Full dataset | Truncated dataset |
| --- | --- | --- |
| Conditional: Morph (Ind) | -0.205 | -0.211 |
| Conditional: Treatment (single) | 0.255 | 0.203 |
| Conditional: MorphInd × Treatment | -0.404 | -0.358 |
| Zero-inflation: Morph (Ind) | 0.665 | 0.745 |
| Zero-inflation: Treatment (single) | -1.073 | -1.018 |
| Zero-inflation: MorphInd × Treatment | 2.455 | 2.376 |
| Key contrast (Ind vs Sat, single EU; OR) | 26.5 | 25.5 |

**Table S17. Comparison of centroid distance model (full vs truncated datasets) – posterior median estimates from brms model**

| Parameter | Full dataset | Truncated dataset |
| --- | --- | --- |
| Intercept | 4.54 | 4.54 |
| Fae–Ind (mixed) | 0.53 | 0.51 |
| Fae–Sat (mixed) | 0.53 | 0.56 |
| Ind–Ind (mixed) | 0.63 | 0.61 |
| Ind–Ind (single) | 0.71 | 0.71 |
| Ind–Sat (mixed) | 0.44 | 0.41 |
